# Exploring the Anti-Alzheimer’s Disease Potential of *Aspergillus terreus* C23-3 Through Genomic Insights, Metabolomic Analysis, and Molecular Docking

**DOI:** 10.3390/jof11080546

**Published:** 2025-07-23

**Authors:** Zeyuan Ma, Longjian Zhou, Zhiyou Yang, Yayue Liu, Yi Zhang

**Affiliations:** 1 Guangdong Provincial Key Laboratory of Aquatic Product Processing and Safety, Guangdong Provincial Engineering Laboratory for Marine Biological Products, Guangdong Provincial Center for Modern Agricultural Scientific Innovation, Shenzhen Institute of Guangdong Ocean University, Zhanjiang Municipal Key Laboratory of Marine Drugs and Nutrition for Brain Health, Research Institute for Marine Drugs and Nutrition, College of Food Science and Technology, Guangdong Ocean University, Zhanjiang 524088, China; gdoumzy@163.com (Z.M.); zhoulongjian@gdou.edu.cn (L.Z.); yang_zhiyou@sina.com (Z.Y.); yayue_liu@163.com (Y.L.); 2 Southern Marine Science and Engineering Guangdong Laboratory (Zhanjiang), Zhanjiang 524088, China; 3 Collaborative Innovation Center of Seafood Deep Processing, Dalian Polytechnic University, Dalian 116034, China

**Keywords:** marine fungi, genome analysis, metabolomic annotation, molecular docking, Alzheimer’s disease

## Abstract

Alzheimer’s disease (AD) is a prevalent neurodegenerative disorder with a pressing need for novel therapeutics. However, current medications only offer symptomatic relief, without tackling the underlying pathology. To explore the bioactive potential of marine-derived fungi, this study focused on *Aspergillus terreus* C23-3, a strain isolated from the coral *Pavona cactus* in Xuwen County, China, which showed a richer metabolite fingerprint among the three deposited *A. terreus* strains. AntiSMASH analysis based on complete genome sequencing predicted 68 biosynthetic gene clusters (BGCs) with 7 BGCs synthesizing compounds reported to have anti-AD potential, including benzodiazepines, benzaldehydes, butenolides, and lovastatin. Liquid chromatography coupled with mass spectrometry (LC-MS)-based combinational metabolomic annotation verified most of the compounds predicted by BGCs with the acetylcholinesterase (AChE) inhibitor territrem B characterized from its fermentation extract. Subsequently, molecular docking showed that these compounds, especially aspulvione B1, possessed strong interactions with AD-related targets including AChE, cyclin-dependent kinase 5-p25 complex (CDK5/p25), glycogen synthase kinase-3β (GSK-3β), and monoamine oxidase-B (MAO-B). In conclusion, the genomic–metabolomic analyses and molecular docking indicated that C23-3 is a high-value source strain for anti-AD natural compounds.

## 1. Introduction

Alzheimer’s disease (AD) is the predominant neurodegenerative disorder and the leading cause of dementia, a condition characterized by progressive cognitive decline with no curative treatments available [1]. The pathogenesis of AD is multifactorial, involving hypotheses such as cholinergic deficit [2], neuroinflammation [3], the hyperphosphorylation of Tau protein [4], and the toxic accumulation of β-amyloid (Aβ) peptides [5]. Recent research has shown that soluble Aβ oligomers (AβOs), generated during the formation of Aβ plaques, exhibit greater toxicity than Aβ itself. These AβOs contribute to synaptic dysfunction, neuroinflammation, and oxidative stress [6]. Current pharmaceutical interventions mainly improve symptoms, failing to address the disease’s underlying pathology [7]. Disease-modifying agents (DMAs) are urgently needed to overcome this severe threat to humans. Fortunately, the exploration of natural products including fungal secondary metabolites (SMs) has led to the discovery of numerous bioactive compounds with neuroprotective potential [8].

Advances in genomics have facilitated the identification of biosynthetic gene clusters (BGCs), enabling the exploration of the untapped chemical diversity within fungal genomes [9]. Increasing novel SMs are being reported by the genome mining of high-value producer strains and successive genetic engineering methods [10,11]. Marine fungi, with adaptations to their unique environment, often exhibit distinct metabolic profiles compared to their terrestrial counterparts [12], rendering them a rich source of novel bioactive compounds. Given the promising biological activities of marine fungal metabolites, they have been positioned as key candidates for developing the next generation of therapeutics [13]. Particularly, marine-derived *Aspergillus*, including many strains of *A. terreus*, has been a prolific source of structurally diverse and biologically active SMs [14].

In our previous studies, several marine *A. terreus* metabolites exhibited anti-AD-related potentials. Among them, butyrolactone I was found to inhibit the production of NO and interleukin-1 β (IL-1β), reduce the expression of inducible nitric oxide synthase (iNOS) and cyclooxygenase-2 (COX-2), inhibit the phosphorylation of nuclear factor kappa-B (NF-κB) in BV2 cells induced by lipopolysaccharide (LPS), and improve the memory of AD model zebrafish injured by AlCl_3_ [15,16]. Two types of benzaldehyde, (*S*)-3-(2,3-dihydroxy-3-methylbutyl)-4-hydroxybenzaldehyde (asperterrol) and 4-hydroxy-3-(3-methyl-2-buten-1-yl)-benzaldehyde, could effectively reduce the secretion of pro-inflammatory factors in mitogen-activated protein kinase (MAPK) pathway in the activated microglia, and thereby improve neuroinflammation. Asperterrol also displayed neuroprotection to Aβ-induced neurological damage in HT-22 cells by inhibiting mitochondrial oxidative stress and blocking neuronal cell apoptosis through Tau protein-related pathways and caspase family-related signaling pathways [17]. Asterrelenin and epi-aszonalenin A, two benzodiazepines, both inhibited Aβ-induced apoptosis in HT-22 cells. Asterrelenin could also effectively inhibit the production of ROS and reduce the expression and secretion levels of GSK-3α/β, CDK5, and p-Tau proteins in neurons [18]. In addition to our study, lovastatin and territrem B, two typical products of *A. terreus*, also have anti-AD activity. It has been reported that lovastatin protects human SK-NSH cells from Aβ-induced apoptosis and reduces the activity of GSK-3β [19], while territrem B is an excellent irreversible inhibitor of AChE [20]. So we believe that *A. terreus* metabolites are highly valuable in the discovery of anti-AD drug leads, and the genome analysis of versatile producer strains may lead to the discovery of more efficient drug candidates.

Mass spectrometry (MS)-based metabolomics like MS-DIAL [21] also play an important role in the mining of natural products by their capability of quickly identifying the chemical components in crude extracts [22]. By similarity matching between the MS spectra of metabolites and the deposited MS spectra, Global Natural Product Social Molecular Networking (GNPS) can speed up the discovery of new natural products, not only identifying even trace amounts of known natural products, but also revealing the internal structural relationship among the metabolites [23,24,25].

In this study, chemical screening of marine *A. terreus* strains identified a fungus *A. terreus* C23-3, whose genome was sequenced for antiSMASH-based BGC prediction targeting anti-AD compounds. Its ability to synthesize relevant compounds was evaluated by LC-MS-based metabolomic annotation. The binding ability of these *A. terreus* metabolites was assessed by molecular docking with multiple targets involved in the AD pathology.

## 2. Materials and Methods

### 2.1. Fungal Fermentation and Secondary Metabolite Analysis

**Figure 1:**
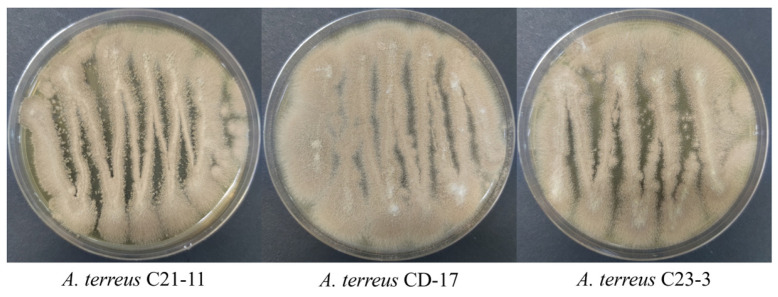
Three strains of marine *A. terreus*.

Three *A. terreus* strains C23-3, C21-11, and CD-17, were previously isolated from the tropical habitats of Zhanjiang seawater, Guangdong, China, between 20°10′36″ and 20°27′00″ N, and 109°50′12″ and 109°56′24″ E (Figure 1, Table 1). Three *A. terreus* strains were grown at 22 °C for 30 days with both 3 g/L and 30 g/L salinity in brown rice medium. To obtain the crude extract of strains, we added an ethyl acetate (EA) and methanol (MeOH) mixed solution (v/v = 3:1) to the medium, conducted supersonic extraction for 30 min, and filtered the solution. This was repeated 3 times, and the supernatant was subjected to rotary evaporation for concentration. The dried sample was redissolved in a dichloromethane (DCM) and MeOH (v/v = 1:1) mixed solvent system, followed by concentration under reduced pressure using a rotary evaporator. Finally, MeOH was used to redissolve the crude extract. The crude extract was diluted to 8 mg/mL for thin-layer chromatography (TLC) analysis: the developer was a DCM and MeOH mixed solution (v/v = 11:1), the thin layer plate was a normal-phase silica gel 60 F254 plate (Qingdao Bangkai High-Tech Materials Co., Ltd., Qingdao, China), and after developing progressed, an ultraviolet reflection transmissometer WFH-201 B (Shanghai Jingke Industry Co., Ltd., Shanghai, China) was used to observe the absorption of different wavelengths (254 nm and 365 nm). The developed plate was used for colorizing displayed by a concentrated sulfuric acid–anisaldehyde reagent.

A total of 10 µL of the sample (8 mg/mL) was injected and eluted with an acetonitrile (ACN)/H_2_O (containing 0.1% HCOOH) gradient with 10% ACN (0–5.0 min), 10–90% ACN (5.0–35.0 min), 90% ACN (35.0–45.0 min), 90–10% ACN (45.0–48.0 min), or 10% ACN (48.0–53.0 min), and the flow rate was 0.6 mL/min on an Agilent 1260 Infinity II on C18 column (Phenomenex Kinetex, 4.6 mm × 100 mm, 5 µm).

### 2.2. Strain Sample Preparation for Sequencing

For genomic DNA extraction and sequencing, strain C23-3 was first inoculated onto potato dextrose agar (PDA) containing 2% salinity and incubated at 28 °C for one week. After a week, it was inoculated in potato dextrose broth (PDB) with 2% salinity and incubated at room temperature for 2 days at 120 rpm. After 2 days, the pellets were collected in a 50 mL centrifuge tube, centrifuged at 4 °C and 5000 rpm, and the medium was removed and the pellets cleaned with sterile saline. The pellets were centrifuged again at 4 °C and 5000 rpm and the liquid was discarded; this was repeated 3 times. Liquid nitrogen was used to freeze the pellets and they were stored at −80 °C until they were used.

### 2.3. Genome Sequencing

Genomic DNA of the *A. terreus* C23-3 was extracted using QIAGEN genomic-tip 100/G columns (QIAGEN, Hilden, Germany) with quality assessment performed through agarose gel electrophoresis and quantified using the NanoDrop 1000 spectrophotometer (Thermo Fisher Scientific). The whole-genome sequencing was performed by BGI Company (Shenzhen, China) through a hybrid strategy combining short-read (DNBSEQ platform) and long-read (PacBio platform) technologies. For DNBSEQ data, SOAPnuke v1.5.6 [26] was employed to filter reads meeting either of the following criteria: (1) reads containing consecutive bases with quality values ≤ 20 accounting for 40% of the total reads, or (2) reads containing N bases exceeding 0.1% of the total, while simultaneously eliminating adapter contamination and duplicate reads. For PacBio data, the processing involved filtering out adapter sequences and subreads shorter than 2000 bp [27,28,29]. High-quality Circular Consensus Sequence (CCS) subreads were generated using ccs-alt v6.4.0 (https://github.com/PacificBiosciences/ccs/releases/tag/v6.4.0, accessed on 20 April 2022), and the filtered data were subsequently assembled with hifiasm v0.17.4-r455 (https://github.com/chhylp123/hifiasm/releases/tag/r455, accessed on 18 December 2022).

### 2.4. BGC Annotation

Secondary metabolite clusters in *A. terreus* C23-3 were computationally predicted using antiSMASH 7.0 with default parameters [30]. The *A. terreus* C23-3 genome was systematically interrogated for secondary metabolite clusters through the hidden Markov model (HMM) profiling of core biosynthetic enzymes, including polyketide synthases (PKSs), non-ribosomal peptide synthetases (NRPSs), terpene cyclases, etc.

### 2.5. Phylogenetic Analysis

Phylogenetic trees were constructed, based on the BGC of butyrolactone I of the target strain and reference strains, by MEGA 11 (Test of Phylogeny: bootstrap method) [31]. A concatenated alignment of conserved biosynthetic enzymes was subjected to bootstrap analysis with the Jukes–Cantor substitution model. A gene cluster comparison figure was drawn by CAGECAT [32]. Reference sequences were retrieved from NCBI GenBank (Table S1).

### 2.6. Molecular Network

The LC-MS/MS data of the extracts of the strain C23-3 were collected on two LC-MS systems. Samples were dissolved in MeOH at 100 µg/mL. All reagents were mass-spectrometry pure.

(1) A total of 10 µL of the sample was injected and eluted with a gradient of H_2_O containing 0.1% HCOOH and ACN (containing 0.1% HCOOH) with a gradient of 30% ACN for 0.5 min, 30–90% ACN for 7.5 min, 90% ACN 3 min, 90–30% ACN for 0.1 min, or 30% ACN for 3.9 min, and a flow rate of 0.3 mL/min on an Orbitrap Fusion Lumos (Thermo Fisher Scientific, Waltham, MA, USA) and a C18 column (Thermo Fisher Scientific-packed Hypersil GOLD, 1.9 µm, 2.1 × 100 mm). Mass spectra were recorded in positive ESI mode (*m*/*z* 50–1500). The obtained raw data were converted into mzXML format and uploaded to GNPS for molecular network construction, and the obtained results were visualized using the software Cytoscape 3.7.2.
(2) Acquity UHPLC DAD Xevo G2-XS Q-Tof liquid chromatography–mass spectrometry instrument (Waters, Milford, MA, USA) was used with an ACN (containing 0.1% HCOOH)/H_2_O (containing 0.1% HCOOH) gradient (30% ACN for 0.5 min, 30–90% ACN for 4.5 min, 90% ACN 3 min, 90–30% ACN for 0.2 min, 30% ACN for 1.3 min) and a flow rate of 0.3 mL/min on a Waters ACQUITY UPLC BEH RP18 column (2.1 × 50 mm, 1.7 µm). Mass spectra were recorded in positive ESI mode (*m*/*z* 50–1500). The obtained raw data were converted into abf format and the chemical composition was analyzed using MS-DIAL.

### 2.7. Molecular Docking

The molecular docking commenced with the structural acquisition and refinement of four neurodegenerative disease-related targets (AChE: 7E3H, CDK5/p25: 7VDP, GSK-3β: 8DJD, MAO-B: 7P4F) from the RCSB PDB database (https://www.rcsb.org/), exclusively selecting X-ray crystallographic structures with resolutions ≤ 2.5 Å (Table S2). The docking pocket was defined based on the binding site of the original ligand, AChE: center_x = −44.88, center_y = 36.781, center_z = −28.92, size_x = 29.25, size_y = 20.25, and size_z = 24.0; CDK5/p25: center_x = 55.191, center_y = −36.612, center_z = 55.655, size_x = 24.0, size_y = 24.0, and size_z = 25.5; GSK−3β: center_x = 5.109, center_y = −1.546, center_z = 33.923, size_x = 26.25, size_y = 21.0, and size_z = 24.75; and MAO-B: center_x = 51.892, center_y = 154.22, center_z = 28.051, size_x = 28.5, size_y = 28.5, and size_z = 27.75.

Receptor pre-processing involved protonation state optimization using AutoDock Tools 1.5.6, including hydrogen addition, heteroatom removal, and Gasteiger–Marsili charge assignment. Concurrently, ligand 3D conformers were generated from SMILES in ChemDraw 22.0, followed by 3D conformational optimization in Chem3D 22.0. Both receptors and ligands were imported into AutoDock Tools 1.5.6 for valence state correction and charge assignment, resulting in standardized .pdbqt formats. The subsequent procedure involved configuring the grid box dimensions and coordinates based on the predicted active site. Using the pre-generated config file, molecular docking was performed with AutoDock Vina 1.2.3 [33]. The top-ranked ligand poses (.pdbqt) were subsequently combined with the receptor file (.pdbqt) and visualized in Pymol 2.5.0; meanwhile, the complexes were exported as .pdb format. Finally, the .pdb files were imported into Discovery Studio 2019 Client for systematic 2D interaction profiling to identify critical hydrogen bonds, hydrophobic contacts, and electrostatic forces.

### 2.8. In Silico Prediction of ADMET and Drug-Likeness Properties

The ADMET properties and drug-likeness characteristics of the compounds were predicted using ADMETlab 3.0 [34], an online bioinformatics platform accessible at https://admetlab3.scbdd.com/.

## 3. Results

### 3.1. Aspergillus terreus Producer Screening

**Figure 2:**
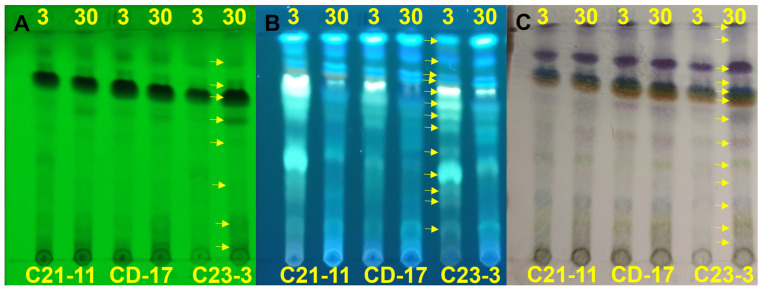
The TLC analysis of *A. terreus* extract. (**A**) Results under UV at 254 nm. (**B**) The fluorescent substances were detected under UV at 365 nm. (**C**) The colorizing result is displayed by concentrated sulfuric acid–anisaldehyde reagent. For (**A**–**C**), the name of the strain (C21-11, CD-17, and C23-3) is at the bottom of the picture; the values at the top of the picture represent the salinity (3 g/L or 30 g/L) during the fermentation. The yellow arrows mark the main metabolites detected by 254 nm UV light, 365 nm UV light, and by H_2_SO_4_-anisaldehyde staining.

**Figure 3:**
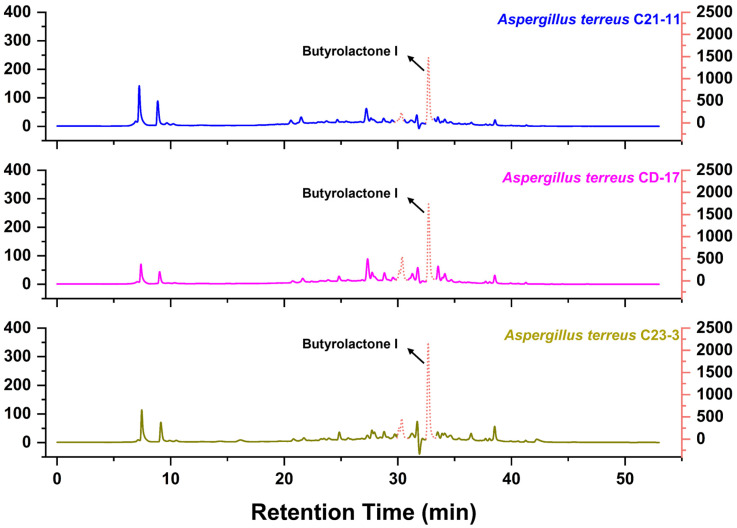
The HPLC analysis of *A. terreus* extract (254 nm). The vertical axis is in milliabsorption units (mAU). The fermentation extracts obtained under a salinity of 3 g/L were used for the analyses. The signal strength of the dotted line is referenced to the right coordinate axis.

**Figure 4:**
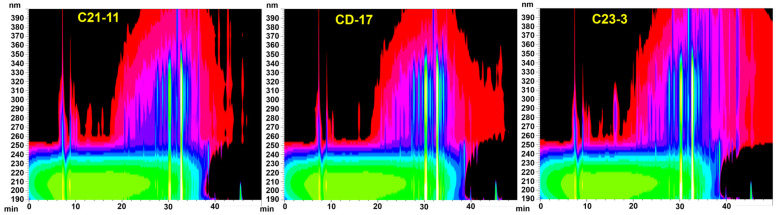
Absorbance–wavelength–time contour plots of *A. terreus* extracts recorded by HPLC-diode array detector (DAD). The fermentation extracts obtained under a salinity of 3 g/L were used for the analyses. The brightness of the color positively correlates with the absorption at specific wavelength.

Three strains of *A. terreus* isolated from different marine habitats were cultured on brown rice medium with two salinity conditions (3 g/L and 30 g/L) and compared for the chemical fingerprints of their crude extracts. TLC analysis (Figure 2) displayed that the three strains had generally similar secondary metabolite profiles, but significant differences were also observed between strains and salinities. The strains cultured under a salinity of 3 g/L produced more fluorescent substances detectable under 365 nm UV light than those under 30 g/L. As is shown (marked with arrow in Figure 2), overall, the strain *A. terreus* C23-3 had more 254 nm UV-active and H_2_SO_4_-anisaldehyde-staining-active main spots when cultured under a salinity of 30 g/L, while it had more 365 nm UV-active main spots when cultured under a salinity of 3 g/L. HPLC analysis of their extracts showed that strain C23-3 also had the highest yield of butylactone I (Figure 3, see butyrolactone I HPLC chromatogram in Figure S1). Figure 4 demonstrates higher UV-active compound content and chemodiversity in strain C23-3’s extract relative to other strains. Thus, by integrating the results from TLC, HPLC, and absorbance–wavelength–time contour plot analyses, strain C23-3 was finally chosen for further investigation on the genome and MS-based metabolome.

### 3.2. Genomic Profile

**Figure 5:**
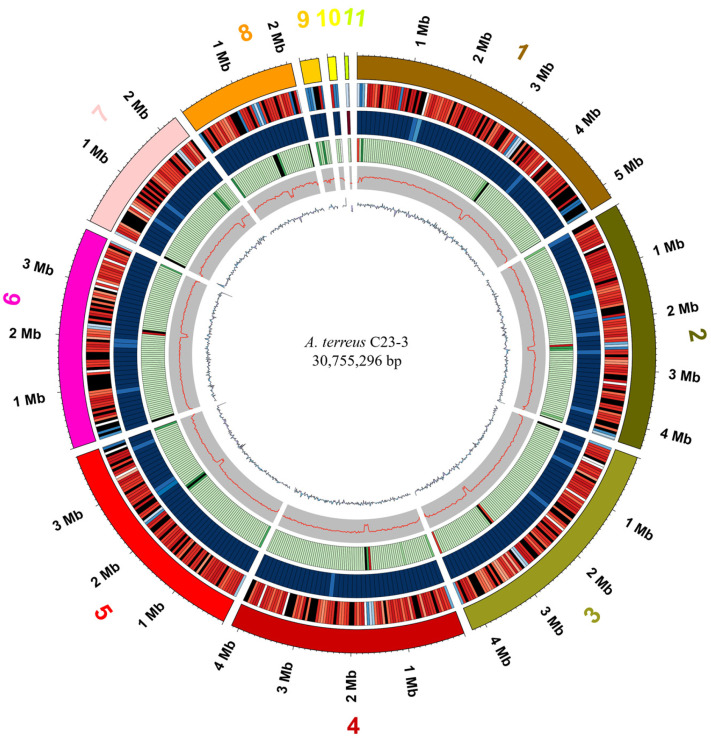
Cycle graph of the *A. terreus* C23-3 genome. From outer to inner circles: 1, Genome (sorted by length); 2, Gene Density (Gene number in 50,000 bp non-overlapping windows); 3, ncRNA Density (ncRNA number in 100,000 bp non-overlapping windows); 4, Repeats Coverage (Repeat_coverage in 50,000 bp non-overlapping windows); 5, GC (GC rate in 20,000 bp non-overlapping windows); 6, GC_skew (GC skew in 20,000 bp non-overlapping windows). The “1–11” around the cycle graph represents the scaffold numbers of the *A. terreus* C23-3 genome.

The de novo genome assembly of *A. terreus* C23-3 generated 11 scaffolds spanning 30,755,296 bp, exhibiting an N50 value of 4,133,560 bp and a GC content of 52.49%. Genome annotation predicted 10,948 protein-coding sequences (CDSs) with a mean length of 1474.61 bp, where 52.47% of CDSs fell within the 500–1499 bp size range (Figure 5; detailed metrics in Figure S2 and Table S3).

### 3.3. BGC Prediction

**Figure 6:**
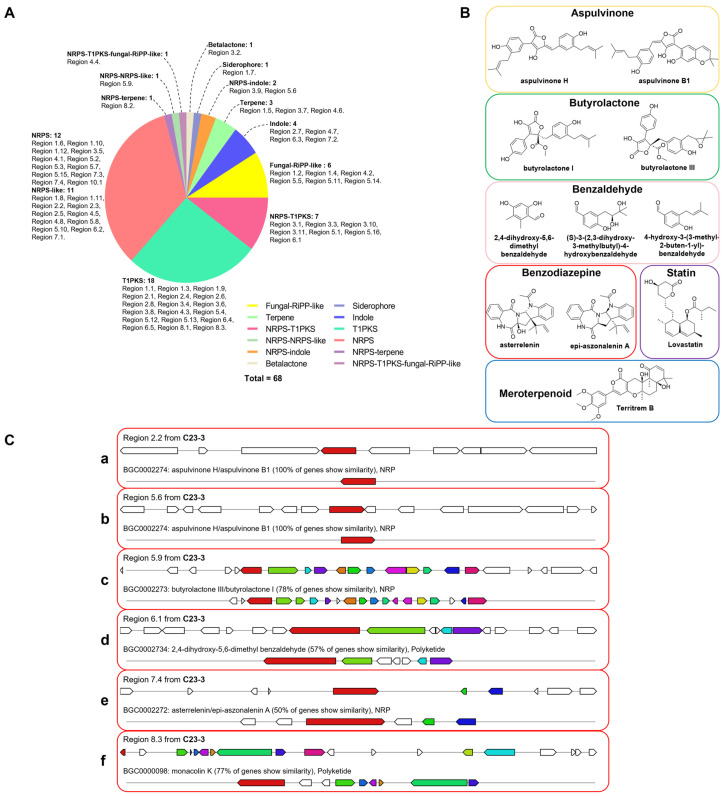
Biosynthetic gene cluster (BGC) prediction in *A. terreus* C23-3 genome, the potential anti-AD compounds reported from this species, and putative BGCs encoding related compounds in the *A. terreus* C23-3 genome. (**A**) Distribution of predicted *A. terreus* C23-3 BGCs of different biosynthetic types. (**B**) Structure of the potential anti-AD compounds from this species. (**C**) Putative *A. terreus* C23-3 BGCs for the potential anti-AD compounds and their homogenous BGCs deposited in the MIBiG database. In each frame, the upper part represents BGCs in *A. terreus* C23-3 and the lower part represents reference BGCs in the MIBiG database.

The 11 scaffolds of *A. terreus* C23-3 were analyzed by antiSMASH, resulting in 68 predicted BGCs (Figure 6A). Based on the previous reports on anti-AD natural products from *A. terreus*, eight compounds (Figure 6B) were linked to six putative BGCs (Figure 6C), including regions 2.2 and 5.6 (aspulvinone BGCs) for aspulvinone H and aspulvinone B1 (Figure 6C(a,b)); region 5.9 (butyrolactone BGC) for butyrolactone I and butyrolactone III (Figure 6C(c)); region 6.1 (benzaldehyde BGC) for 2,4-dihydroxy-5,6-dimethyl benzaldehyde, an analog of (*S*)-3-(2,3-dihydroxy-3-methylbutyl)-4-hydroxybenzaldehyde and 4-hydroxy-3-(3-methyl-2-buten-1-yl)-benzaldehyde with anti-AD potential (Figure 6C(d)); region 7.4 (benzodiazepine BGC) for asterrelenin and epi-aszonalenin A (Figure 6C(e)); and region 8.3 (statin BGC) for lovastatin/monacolin K (Figure 6C(f)).

**Figure 7:**
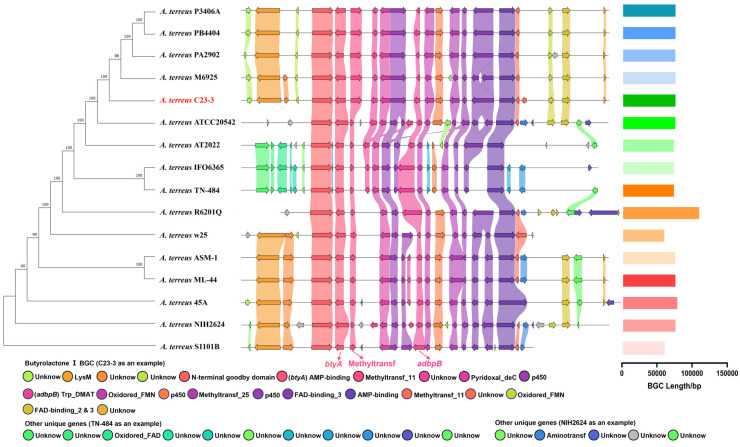
Phylogenetic tree based on the BGC of butyrolactone I. The maximum-likelihood tree was reconstructed using homologous BGC sequences predicted by antiSMASH from 16 *A. terreus* genomes. The bar chart to the right of the image represents the lengths of individual BGCs (bp). Beneath the figure, annotations corresponding to individual components of the BGC are displayed in a left-to-right orientation.

Comparative analysis of butyrolactone I BGC across *A. terreus* strains revealed conserved secondary metabolite biosynthesis mechanisms at the intraspecies level. Genome mining via antiSMASH 7.0 identified homologous BGCs in 16 *A. terreus* genomes, exhibiting remarkable structural conservation (Figure 7). Phylogenomic reconstruction using the maximum-likelihood methodology demonstrated that strain *A. terreus* C23-3 clustered most closely with M6925. Quantitatively, these BGCs exhibited length conservation (mean ± SD: 78,227 ± 10,961 kb, n = 16). In the previous reports on the biosynthetic mechanism of butyrolactone I in *A. terreus*, core biosynthetic components included the following: (1) *btyA* encoding the essential NRPS-like synthase; (2) methyltransferase transferring the methyl group of S-adenosylmethionine (SAM) to the substrate; and (3) *adbpB* encoding the prenyltransferase for isoprenoid side-chain addition [35,36]. Strain-specific variations primarily occurred in genetic elements flanking the core biosynthetic genes, encompassing possible tailoring enzyme genes like methyltransferases (e.g., Methyltransf 11), oxidoreductases (e.g., Oxidored FMN, p450, FAD binding protein), and others. These genetic elements likely mediate structural modifications, driving strain–specific chemical diversification.

### 3.4. Metabolomic Analysis

**Figure 8:**
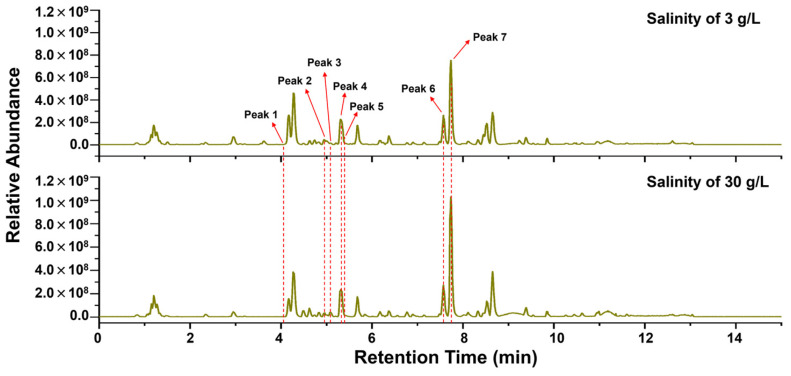
BPC of strain C23-3’s fermentation extracts.

**Figure 9:**
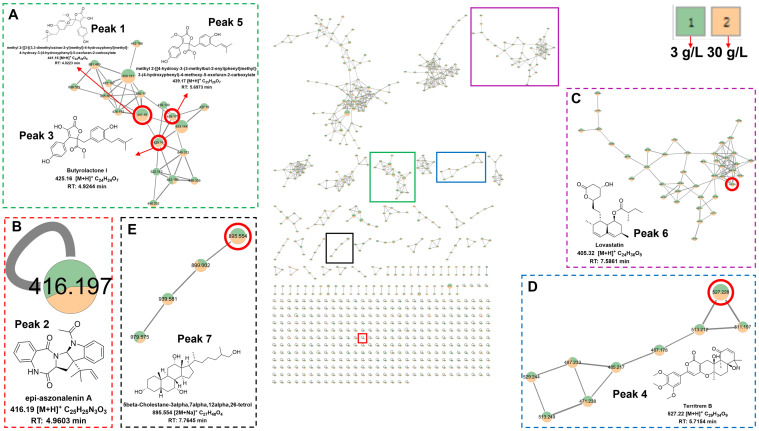
GNPS molecular network with its partially enlarged diagrams for the metabolites of strain C23-3 constructed on the basis of MS^2^ relationships. “1” represents a salinity of 3 g/L at the fermentation; “2” represents a salinity of 30 g/L at the fermentation. (**A**) Network of butyrolactones. (**B**) Node of epi-aszonalenin A. (**C**) Network of statins. (**D**) Network of territrems. (**E**) Network of 5β-Cholestane-3α,7α,12α,26-tetrol derivatives.

The fermentation extract of C23-3 (cultured under a salinity of 3 g/L and 30 g/L) was further analyzed by LC-MS/MS (Figure 8). The base peak chromatogram (BPC) demonstrated the higher intensity of C23-3’s metabolite peak 7 under a salinity of 30 g/L than 3 g/L. And the data were further annotated by GNPS and MS-DIAL (Table S4). As a result, peak 7 was annotated as 5β-Cholestane-3α,7α,12α,26-tetrol by GNPS (see MS and MS^2^ spectra in Figure S3 and MS^2^ mirror-matching spectra in Figure S4). In addition, GNPS directly annotated six compounds with previous anti-AD activity reports, including butyrolactone I, methyl-2-[[3-[(3,3-dimethyloxiran-2-yl)methyl]-4-hydroxyphenyl]methyl]-4-hydroxy-3-(4-hydroxyphenyl)-5-oxofuran-2-carboxylate, methyl-2-[[4-hydroxy-3-(3-methylbut-2-enyl)phenyl]methyl]-3-(4-hydroxyphenyl)-4-methoxy-5-oxofuran-2-carboxylate, epi-aszonalenin A, lovastatin, and territrem B (Figure 9; see MS^2^ mirror-matching spectra in Figure S4). Of these, butyrolactone I, epi-aszonalenin A, and lovastatin were antiSMASH-predicted compounds, while the other predicted compounds, 2,4-dihydroxy-5,6-dimethyl benzaldehyde, aspulvinone B1, aspulvinone H, butyrolactone III, and asterrelenin, were not annotated in this metabolomic analysis. The above seven compounds also contained rich congeners as their neighbor nodes in the clusters, especially for statins and butyrolactones, revealing their versatility in these types of useful metabolites and the possibility of new compounds.

**Figure 10:**
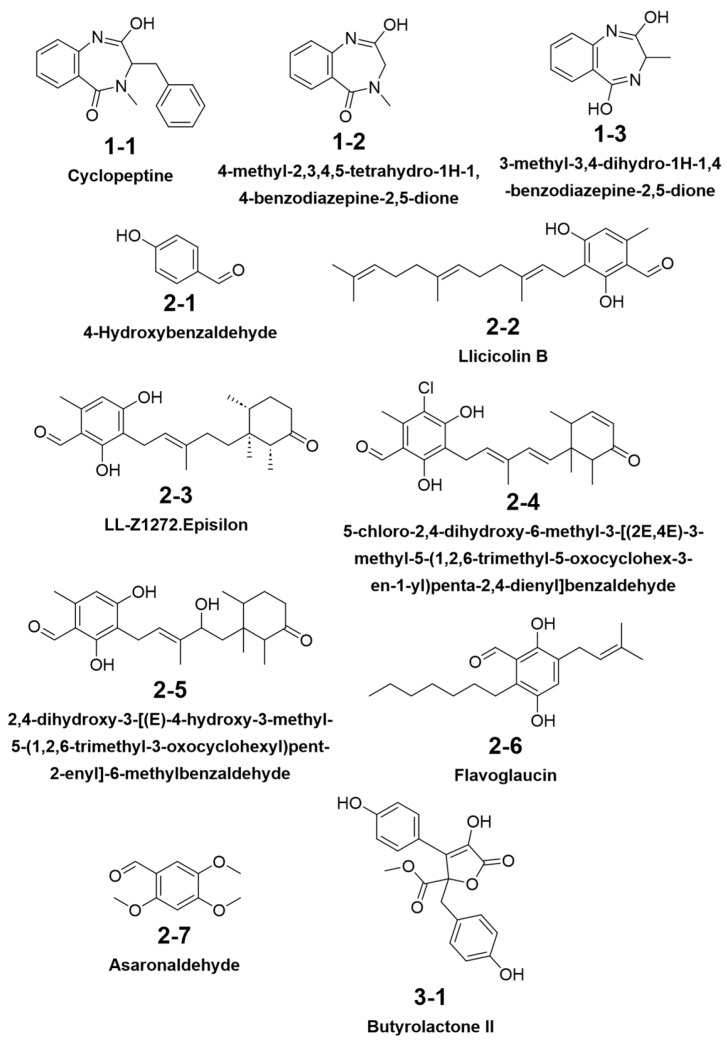
Structure of MS-DIAL annotation results.

MS-DIAL annotated additional types of compounds of interest with acceptable MS similarity and biological resources (Figure 10; see MS^2^ mirror-matching spectra in Figure S5). Compounds 1-1, 1-2, and 1-3 are benzodiazepines with the precursors anthranilic acid and another amino acid, and were previously reported as the metabolites of *Penicillium claviforme* [37]. Benzaldehydes are common fungal secondary metabolites [38]. Compounds 2-1 to 2-7 share a benzoaldehyde ring with ortho- or para- oxygenated substitution, among which 2-2 to 2-5 are meroterpenes containing similar linear or cyclized monoterpene modules. The biological resources of compounds 2-2 and 2-3 were previously reported from *Fusarium* sp. [39] and *Acremonium* sp. [40]; 2-1 and 2-5 to 2-7 were also reported to have been derived from fungi [41,42,43,44]; this supports the reasonability of their presence in *A. terreus* C23-3 extract. Compound 3-1 is butyrolactone II with reports from *A. terreus* [45].

### 3.5. Molecular Docking Analysis

**Figure 11:**
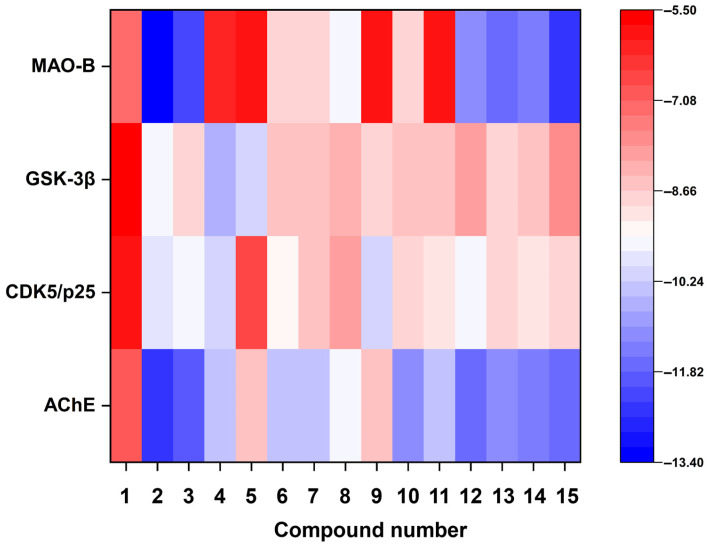
Binding energy between 15 compounds and AD targets. The X-axis is assigned to compound names and the Y-axis is annotated with AD targets. A color key (right panel) displays the values ranging from −13.40 (dark blue) to −5.50 (bright red).

The multifactorial pathogenesis of AD involves the dysregulation of key enzymatic targets, including AChE (impairment of neurotransmitter acetylcholine and Aβ aggregation), CDK5/p25 (Tau hyperphosphorylation), GSK-3β (neurofibrillary tangle formation), and MAO-B (H_2_O_2_-mediated oxidative stress and Aβ/Tau pathology crosstalk) [46,47,48,49]. Molecular docking analysis of 11 metabolites derived from *A. terreus* (Table 2, annotated by antiSMASH and GNPS in this study) identified aspulvinone B1 as a multi-targeted binder with favorable binding energies across all the tested targets (−9.5 to −13.4 kcal/mol). Notably, aspulvinone B1′s binding energies against all critical targets were lower than the binding energies of all positive controls to the corresponding AD targets. It demonstrated the lowest binding energies against two critical targets, AChE (−12.4 kcal/mol) and MAO-B (−13.4 kcal/mol), and low binding energies to CDK5/p25 (−9.9 kcal/mol) and GSK-3β (−9.5 kcal/mol) as well (Figure 11).

**Figure 12:**
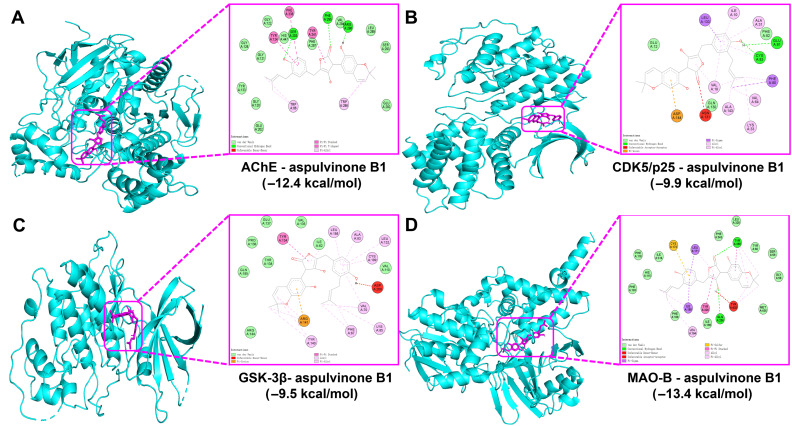
Interaction diagram of molecular docking of aspulvinone B1 and key AD targets. (**A**) Docking with AChE. (**B**) Docking with CDK5/p25 complex. (**C**) Docking with GSK-3β. (**D**) Docking with MAO-B.

In the AChE–aspulvinone B1 complex, the stable binding is primarily mediated by hydrogen bonds with Ser203, Phe295, and Arg296, and hydrophobic interactions with residues such as Trp86, Trp286, and Tyr124 (Figure 12).

For the complex of aspulvinone B1 and CDK5/p25, key interactions include hydrogen bonds with Glu81 and Cys83, and hydrophobic interactions with key residues such as Phe80, Leu133, and Ile10, supplemented by an electrostatic π anion interaction with the Asp144 (Figure 12).

In the GSK-3β binding pocket, aspulvinone B1 establishes hydrophobic interactions with several residues such as Phe67, Val70, and Ala83 (Figure 12).

In the MAO-B binding pocket, aspulvinone B1 forms hydrogen bonds with Gln206 and Tyr398, and hydrophobic interactions with multiple residues including Leu164, Leu171, and Ile199 (Figure 12).

The docking results for other compounds are shown in Figures S6–S13. Aspulvinone H, the two benzodiazepines, and territrem B also displayed polyhedral and strong interactions with anti-AD targets.

Overall, the 11 *A. terreus* SMs bind to AChE mainly through hydrophobic interactions and hydrogen bonding (Table S5).

Their bindings to the CDK5/p25 complex are mainly through a large number of hydrophobic interactions and hydrogen bonds with the acidic residues (Glu and Asp). Some compounds further stabilize their complexes with this target by electrostatic interactions with the Asp residues (Table S6).

Their bindings to GSK-3β are mainly through hydrophobic interactions. Among the compounds, asterrelenin and aspulvinone B1 form the largest number of hydrophobic interactions. Notably, aspulvinone B1 exhibits low binding energy and two benzodiazepines achieve the lowest binding energies with GSK-3β. The benzodiazepines additionally form hydrogen bonds with Gln185 alongside hydrophobic interactions, and this combination makes the binding more stable (Table S7).

In docking with MAO-B, the binding conformation is mainly stabilized through hydrophobic interactions and hydrogen bonds (Table S8).

### 3.6. ADMET Prediction

To evaluate the pharmaceutical potential of these compounds, their ADMET properties and drug-likeness characteristics were predicted utilizing the ADMETlab 3.0 platform (Tables S9–S17). Butyrolactone I and lovastatin were excluded as they have been previously characterized in the literature [16,50]. Overall, the tested compounds demonstrated generally acceptable ADMET profiles and drug-likeness traits. Taking aspulvinone B1 and epi-aszonalenin A as representative examples, both compounds exhibited effective human intestinal absorption (HIA), accompanied by optimal Caco-2 permeability, and an appropriate steady-state volume of distribution. In terms of safety assessment, these two compounds performed satisfactorily across key metrics, including hERG blockade and carcinogenicity. Furthermore, they complied with critical drug-likeness criteria such as the Lipinski rule [51], Pfizer rule [52], and golden triangle principle [53], collectively validating their potential as drug candidates. Unfortunately, aspulvinone B1 and epi-aszonalenin A also displayed certain limitations: both exhibited poor blood–brain barrier (BBB) penetration, an elevated risk of human hepatotoxicity, and the potential to induce liver injury.

## 4. Discussion

Strain C23-3 was isolated from the coral *Pavona cactus* collected in Zhanjiang, China. Preliminary pharmacological screening revealed that multiple SMs derived from C23-3 exhibited promising anti-AD activity. Additionally, initial screening revealed that its crude extract exhibited the highest AChE inhibitory activity among the tested marine fungi [54]; this finding collectively demonstrated its potential as a prolific source strain of novel anti-AD therapeutic agents. In addition, C23-3 has been previously cultured by chemical induction and co-culture [55], showing its capability of synthesizing rich secondary metabolites under stimulus. To systematically evaluate their biosynthetic potential, three marine-derived *A. terreus* strains including C23-3 were analyzed by comparative metabolomics. The results demonstrated that C23-3 possessed significantly greater metabolic diversity, particularly in specialized metabolite production, highlighting its exceptional secondary metabolic capacity. Building on these chemical findings, we performed whole-genome sequencing to elucidate the genetic basis of its metabolic versatility. This genomic investigation aimed to systematically characterize its biosynthetic gene clusters, establish genotype–phenotype correlations between genetic architecture and observed chemical profiles, and provide a comprehensive genetic blueprint for a future exploration of its pharmacological potential.

Whole-genome sequencing revealed that *A. terreus* C23-3 possesses a 30.76 Mb genome organized into 11 scaffolds. To elucidate biosynthetic potential, strain C23-3’s BGCs were annotated by antiSMASH. A total of 68 BGCs were predicted and the genome harbors numerous NRPSs, NRPS-like BGCs, PKSs, and other BGCs, including hybrid ones, revealing the metabolic diversity of the secondary metabolites in strain C23-3. This genomic novelty suggests C23-3 possesses the capacity to produce structurally distinct secondary metabolites. The genomes of two marine *A. terreus* strains (M7 [56] and B12 [57]) were analyzed with antiSMASH, predicting 75 and 67 BGCs, respectively. And the numbers of NRPS BGCs in both M7 (28) and B12 (27) exceed that in strain C23-3 (23). Beyond NRPSs, strain M7 possesses more PKS BGCs (22) than strain C23-3 (18), whereas B12 has fewer PKS BGCs (17). Additionally, strain C23-3 has more NRPS-PKS hybrid BGCs (7) than both M7 (6) and B12 (5). Furthermore, two unpredicted scaffolds were identified in C23-3. These findings revealed strain C23-3’s unique potential in producing secondary metabolites. Traditional methods for natural product discovery are time-consuming and inefficient, often facing the challenge of a high rediscovery rate. Genome mining, utilizing bioinformatics tools to predict BGCs, significantly accelerates this process [58]. Furthermore, genome mining integrated with metabolomics can correlate predicted BGCs with the SMs which are actually produced, thereby further improving the efficiency of bioactive compounds’ discovery [59]. The GNPS-plus-MS-DIAL annotation verified the capability of strain C23-3 to synthesize the potential anti-AD compounds of the structural types predicted by antiSMASH (butyrolactone I, epi-aszonalenin A, and lovastatin), correlating antiSMASH-predicted BGCs with the actual SMs produced. We also noticed that not all the predicted compounds had been detected or annotated in a metabolomics survey, which may be due to the limited MS^2^ spectral libraries of GNPS and MS-DIAL or the silence of BGC expression under the employed culture condition. More diverse cultural strategies like OSMAC (One Strain Many Compounds) or rational genome mining methods like CRISPRa can be used to explore more active compounds from this strain.

The molecular docking analysis firstly reveals the binding affinity of four butenolides to all of the four AD targets. Among them, α,γ-type butenolides (aspulvinones B1 and H) exhibited an overall higher affinity to the targets than β,γ-type butenolides (butyrolactones I and III) and the other compounds. In particular, apsulvinone B1 displayed binding energies of −9.5 to −13.4 kcal/mol to all the four targets. This is consistent with the tendency shown in a previous docking study in which aspulvinone H, aspulvinone B1, butyrolactone I, and butyrolactone III displayed binding energies with MAO-B (PDB ID: 2V5Z, Resolution: 1.60 Å) of −10.3 kcal/mol, −9.6 kcal/mol, −7.5 kcal/mol, and −7.9 kcal/mol, respectively [60,61]. Furthermore, the two α,γ-type butenolides also showed higher affinity with MAO-B than AChE. This is also in line with a previous report on aspulvinone H, in which it demonstrated target selectivity with the strongest binding affinity for MAO (−11.9 kcal/mol) compared to AChE (−10.6 kcal/mol) [62]. These indicate the potential of α,γ-butenolides as multi-target anti-AD agents, especially as MAO-B inhibitors. Apart from that, aspulvinone H has favorable DPPH-scavenging activity [63] as well as moderate AChE inhibitory activity [64].

Butyrolactone I also demonstrated favorable binding energies with AChE, CDK5/p25, GSK-3β, and MAO-B (−10.3 kcal/mol, −9.2 kcal/mol, −8.4 kcal/mol, and −8.9 kcal/mol, respectively). It has been shown that butyrolactone I has CDK1 and CDK5 inhibitory activity [65], and is able to inhibit Tau phosphorylation by inhibiting CDK5 [66]. In another study, it showed an IC_50_ of 0.43 µM and 0.077 µM for CDK1/cyclin B and CDK5/p25, respectively, whereas its derivative butyrolactone I 3-sulfate inhibited these two targets more than 20-fold [67]. It has also been reported that butyrolactone I not only exerts inhibitory effects on CDK5 but also has GSK-3β inhibitory activity [68]. By the gold nanoparticle (GNP) screening method, butyrolactone I and a benzaldehyde were found to moderate anti-Aβ aggregation activity, suggesting that they may be potential Aβ aggregation inhibitors (AAIs) [69]. The occurrence of colitis exacerbates the condition of AD [70], and butyrolactone I can play a role in alleviating colitis in mice [71], which can alleviate the condition of AD to a certain extent. Recent findings indicate that the intragastric administration of butyrolactone I improves cognitive deficits caused by intracerebroventricular Aβ_1-42_ injection in mice, while concurrently maintaining the gut microbiota balance, enhancing short-chain fatty acid levels. It can also reverse the Aβ_1-42_-induced activation of hippocampal microglia and astrocytes, suppressing the elevation of oxidative stress and pro-inflammatory cytokines in both plasma and the brain [72]. Combining the results of this molecular docking experiment and the existing studies, the potential of β,γ-type and α,γ-type butenolides to prevent neurodegenerative diseases is expected to undergo in-depth investigation.

Lovastatin, while primarily recognized for its cardiovascular benefits, displays dual anti-AD properties: neuronal protection during disease progression [73] and Aβ reduction [74,75]. Epidemiological studies indicate that lovastatin intake reduces the risk of dementia [76]. Furthermore, lovastatin can upregulate α7nAChR (nicotinic acetylcholine receptor) expression, decrease cholinesterase activity, and reduce αAPP accumulation, suggesting its potential neuroprotective role in AD treatment [77]. Lovastatin has been shown to reduce Aβ production [78], as statins specifically modulate the maturation and phosphorylation of amyloid precursor protein (APP) in cultured neurons, resulting in decreased Aβ generation [79]. Our docking results align with its AChE inhibitory capacity (with binding energy of −11.2 kcal/mol) and also show its interaction with another neurotransmitter-degrading enzyme, MAO-B (−8.9 kcal/mol). In the present molecular docking experiment, lovastatin also exhibited a favorable binding energy (−8.4 kcal/mol) with GSK-3β and CDK5/p25 (−8.9 kcal/mol). Research indicates that lovastatin protected neuronal cells from Aβ-induced apoptosis and reduced GSK-3β activity [19]. This inhibition of GSK-3β and CDK5 can additionally reduce Tau phosphorylation [80,81].

Territrem B, as a non-covalent irreversible AChE inhibitor, is hypothesized to mechanically anchor within the enzyme’s active gorge [82]. Our docking results corroborate this mechanism, showing robust hydrophobic engagement with Trp86, Tyr124, and Phe297 and π-π interactions involving π-π stacked with Trp286, Tyr341, and Phe338. Both these interactions stabilize the bound conformation. Furthermore, it displays low binding energies (−9.0 kcal/mol and −8.5 kcal/mol) with CDK5/p25 and GSK-3β, which have not been reported before to our knowledge.

Two benzodiazepines (asterrelenin and epi-aszonalenin A) exhibited the lowest binding energies (−10.7 kcal/mol and −10.1 kcal/mol, respectively) with GSK-3β when compared to the other compounds. This finding suggests their new potential as GSK-3β inhibitors, highlighting their promising applicability as novel therapeutic candidates for AD intervention. In addition, epi-aszonalenin A can downregulate NF-κB expression and nuclear translocation, indicating that it also has anti-inflammatory activity [83].

The above docking analysis conducted in this study revealed the considerable prospect of *A. terreus* metabolites, especially aspulvinone B1, for treating AD. Given that α,γ-type butenolides like aspulvinone B1 are absent in reports on anti-AD effects and mechanisms and are not detected/annotated in the extract of *A. terreus* C23-3, they are worthy of further study on BGC activation and following anti-AD tests. In addition, as a routine metabolite of strain C23-3, epi-aszonalenin A‘s excellent binding affinity with GSK-3β arouses further interest to verify its in vitro and in vivo inhibitory activity regarding Aβ accumulation and the hyperphosphorylation of Tau protein.

Although a variety of potential compounds were predicted by antiSMASH from *A. terreus* C23-3’s genome or annotated through MS-based metabolomic analysis, complete and clear connections between genomics and metabolomics are still needed to explore their biosynthetic ability. Some of the predicted or annotated metabolites remain to be verified by further compound isolation and purification in BGC activation studies using different strategies like OSMAC and pathway-specific genome mining methods (like CRISPRa, pathway-specific transcription factor over-expression, and heterogeneous expression) or pathway non-specific methods (like epigenetic modification and global transcription factor overexpression). In addition, the molecular docking analysis revealed that the anti-AD potential of these compounds should be further validated to determine their biological activities by in vitro and in vivo experiments. For example, preliminary validation of the compounds’ anti-AD activity will be carried out through in vitro cellular assays using neuronal system-associated cell models. Subsequent in vivo investigations employing established AD animal models and suitable drug delivery routes will further explore their anti-AD efficacy and underlying mechanisms.

## 5. Conclusions

Overall, genomic, metabolomic, and molecular docking analyses collectively indicated *A. terreus* C23-3 as a high-value source strain for future studies pursuing diverse novel anti-AD compounds and probing into their mechanisms of action. The genomics–metabolomics linkage requires further elucidation to fully unlock *A. terreus* C23-3’s biosynthetic potential. The predicted metabolites need to be isolated and elucidated through comprehensive chemical investigation propelled by OSMAC, CRISPRa, heterologous expression, and epigenetic modification, and further verified for their bioactivities by in vitro/vivo experiments.

## Figures and Tables

**Table 1:** Information on three strains of marine *A. terreus*.

Strain	Isolation Source	Depository Institution	Accession Number	GenBank Number
C21-11	*Porites pukoensis*	GDMCC	GDMCC 62180	JQ717316
CD-17	*Holothuria scabra*	Laboratory	GDOUMDI2021CD17	OM319845.1
C23-3	*Pavona cactus*	GDMCC	GDMCC 60316	MG707631.1

GDMCC: Guangdong Microbial Culture Collection Center.

**Table 2:** The compounds screened by molecular docking against AD targets.

No	Name	Structural Type (Method of Annotation)
1	2,4-dihydroxy-5,6-dimethyl benzaldehyde	benzaldehyde (annotated by antiSMASH)
2	aspulvinone B1	β,γ-type butenolide (annotated by antiSMASH)
3	aspulvinone H	β,γ-type butanolide (annotated by antiSMASH)
4	asterrelenin	benzodiazepine (annotated by antiSMASH)
5	epi-aszonalenin A	benzodiazepine (co-annotated by antiSMASH and GNPS)
6	butyrolactone I	α,γ-type butanolide (co-annotated by antiSMASH and GNPS)
7	butyrolactone III	α,γ-type butanolide (annotated by antiSMASH)
8	BTL-A	α,γ-type butanolide (annotated by GNPS)
9	BTL-B	α,γ-type butanolide (annotated by GNPS)
10	lovastatin	statin (co-annotated by antiSMASH and GNPS)
11	territrem B	meroterpenoid (annotated by GNPS)
12	donepezil	positive control of AChE
13	P_CDK5/p25	positive control of CDK5/p25
14	P_GSK-3β	positive control of GSK-3β
15	P_MAO-B	positive control of MAO-B

BTL-A is methyl-2-[[3-[(3,3-dimethyloxiran-2-yl)methyl]-4-hydroxyphenyl]methyl]-4-hydroxy-3-(4-hydroxyphenyl)-5-oxofuran-2-carboxylate; BTL-B is methyl-2-[[4-hydroxy-3-(3-methylbut-2-enyl)phenyl]methyl]-3-(4-hydroxyphenyl)-4-methoxy-5-oxofuran-2-carboxylate; P_CDK5/p25 is [1-[3-fluoranyl-4-[(2-piperidin-4-yloxy-1,6-naphthyridin-7-yl)amino]phenyl]pyrazol-3-yl]methanol; P_GSK-3β is 2-[(cyclopropanecarbonyl)amino]-N-(5-phenylpyridin-3-yl)pyridine-4-carboxamide; P_MAO-B is 4-(hydroxymethyl)-7-[[4-[[methyl-(phenylmethyl)amino]methyl]phenyl]methoxy]chromen-2-one.

## Data Availability

Data are contained within the article and Supplementary Materials.

## Supplementary Materials

The following supporting information can be downloaded at: https://www.mdpi.com/article/10.3390/jof11080546/s1, Figure S1: The HPLC analysis of *A. terreus* extract and butylactone I; Figure S2: Length of *A. terreus* C23-3’s CDS; Figure S3: MS and MS^2^ spectrogram of peak 7; Figure S4: MS^2^ mirror-matching spectra of GNPS annotation; Figure S5: MS^2^ mirror-matching spectra of MS-DIAL annotation; Figure S6: Target compound docking with AChE (except aspulvinone B1); Figure S7: Positive control docking with AChE; Figure S8: Target compound docking with CDK5/p25 (except aspulvinone B1); Figure S9: Positive control docking with CDK5/p25; Figure S10: Target compound docking with GSK-3β (except aspulvinone B1); Figure S11: Positive control docking with GSK-3β; Figure S12: Target compound docking with MAO-B (except aspulvinone B1); Figure S13: Positive control docking with MAO-B; Table S1: GenBank ID of reference sequences in phylogenetic trees; Table S2: Information regarding proteins’ structural acquisition from the RCSB PDB database; Table S3: Statistical assembly of the *A. terreus* C23-3 genome; Table S4: GNPS and MS-DIAL annotation results; Table S5: Result of docking with AChE; Table S6: Result of docking with CDK5/p25; Table S7: Result of docking with GSK-3β; Table S8: Result of docking with MAO-B; Table S9: ADMET and drug-likeness properties of 2,4-dihydroxy-5,6-dimethyl benzaldehyde determined through the online prediction tool ADMETlab 3.0; Table S10: ADMET and drug-likeness properties of aspulvinone B1 determined through the online prediction tool ADMETlab 3.0; Table S11: ADMET and drug-likeness properties of aspulvinone H determined through the online prediction tool ADMETlab 3.0; Table S12: ADMET and drug-likeness properties of asterrelenin determined through the online prediction tool ADMETlab 3.0; Table S13: ADMET and drug-likeness properties of epi-aszonalenin A determined through the online prediction tool ADMETlab 3.0; Table S14: ADMET and drug-likeness properties of butyrolactone III determined through the online prediction tool ADMETlab 3.0; Table S15: ADMET and drug-likeness properties of BTL-A determined through the online prediction tool ADMETlab 3.0; Table S16: ADMET and drug-likeness properties of BTL-B determined through the online prediction tool ADMETlab 3.0; Table S17: ADMET and drug-likeness properties of territrem B determined through the online prediction tool ADMETlab 3.0.

## Abbreviations

The following abbreviations are used in this manuscript:

AD	Alzheimer’s disease
BGCs	Biosynthetic gene clusters
LC-MS	Liquid chromatography coupled with mass spectrometry
AChE	Acetylcholinesterase
CDK5/p25	Cyclin-dependent kinase 5-p25 complex
GSK-3β	Glycogen synthase kinase-3β
MAO-B	Monoamine oxidase-B
Aβ	β-amyloid
AβOs	Aβ oligomers
DMAs	Disease-modifying agents
SMs	Secondary metabolites
IL-1β	Interleukin-1 β
iNOS	Inducible nitric oxide synthase
COX-2	Cyclooxygenase-2
NF-κB	Nuclear factor kappa-B
LPS	Lipopolysaccharide
MAPK	Mitogen-activated protein kinase
MS	Mass spectrometry
GNPS	Global natural product social molecular networking
EA	Ethyl acetate
MeOH	Methanol
DCM	Dichloromethane
TLC	Thin-layer chromatography
ROI	Rectangular region of interest
ACN	Acetonitrile
PDA	Potato dextrose agar
PDB	Potato dextrose broth
CCS	Circular consensus sequence
HMM	Hidden Markov model
PKS	Polyketide synthase
NRPS	Non-ribosomal peptide synthetase
CDS	Coding sequence
SAM	S-adenosylmethionine
BPC	Base peak chromatogram
HIA	Human intestinal absorption
BBB	Blood–brain barrier
OSMAC	One strain many compounds
GNP	Gold nanoparticle
AAIs	Aβ aggregation inhibitors
nAChR	Nicotinic acetylcholine receptor
APP	Amyloid precursor protein
IDE	Insulin-degrading enzyme
