# Supplementary material for: Exploring the Anti-Alzheimer’s Disease Potential of Aspergillus terreus C23-3 Through Genomic Insights, Metabolomic Analysis, and Molecular Docking
Source: J Fungi (Basel). 2025 Jul 23;11(8):546. doi: 10.3390/jof11080546 (PMC12387979; doi:10.3390/jof11080546)
Supplement: Supplementary file 1 [file jof-11-00546-s001.zip › jof-3677653-supplementary.pdf]

Table S1. GenBank ID of reference sequences in phylogenetic trees.

Strain	Gene Bank ID	Assembly
A. terreus P3406A	GCA_023625495.1	ASM2362549v1
A. terreus PB4404	GCA_023625535.1	ASM2362553v1
A. terreus PA2902	GCA_023625575.1	ASM2362557v1
A. terreus M6925	GCA_009834425.1	ASM983442v1
A. terreus ATCC20542	GCA_016808415.1	ASM1680841v1
A. terreus AT2022	GCA_034642025.1	ASM3464202v1
A. terreus IFO6365	GCA_009932835.1	Ater_IFO6365_01
A. terreus TN-484	GCA_009014675.2	Ater_TN-484_02
A. terreus R6201Q	GCA_037041815.1	ASM3704181v1
A. terreus w25	GCA_002749855.1	ASM274985v1
A. terreus ASM-1	GCA_015266375.1	ASM1526637v1
A. terreus ML-44	GCA_015333565.1	ASM1533356v1
A. terreus 45A	GCA_001630395.1	ASM163039v1
A. terreus NIH2624	GCA_000149615.1	ASM14961v1
A. terreus S1101B	GCA_023625515.1	ASM2362551v1

Table S2. Information of proteins structural acquisition from the RCSB PDB database.

Target	Resolution	PDB ID	Positive control	SMILES of positive control
AChE	2.45 Å	7E3H	Donepezil	COC1=C(C=C2C(=C1)CC(C2=O)CC3CCN(CC3)CC4=CC=CC=C4)OC
CDK5/p25	2.09 Å	7VDP	[1-[3-fluoranyl-4-[(2-piperidin-4-yloxy-1,6-naphthyridin-7-yl)amino]phenyl]pyrazol-3-yl]methanol	C1CNCCC1OC2=NC3=CC(=NC=C3C=C2)NC4=C(C=C(C=C4)N5C=CC(=N5)CO)F
GSK-3β	2.21 Å	8DJD	2-[(cyclopropanecarbonyl)amino]-N-(5-phenylpyridin-3-yl)pyridine-4-carboxamide	C1CC1C(=O)NC2=NC=CC(=C2)C(=O)NC3=CN=CC(=C3)C4=CC=CC=C4
MAO-B	2.30 Å	7P4F	4-(hydroxymethyl)-7-[[4-[[methyl-(phenylmethyl)amino]methyl]phenyl]methoxy]chromen-2-one	CN(CC1=CC=CC=C1)CC2=CC=C(C=C2)COC3=CC4=C(C=C3)C(=CC(=O)O4)CO

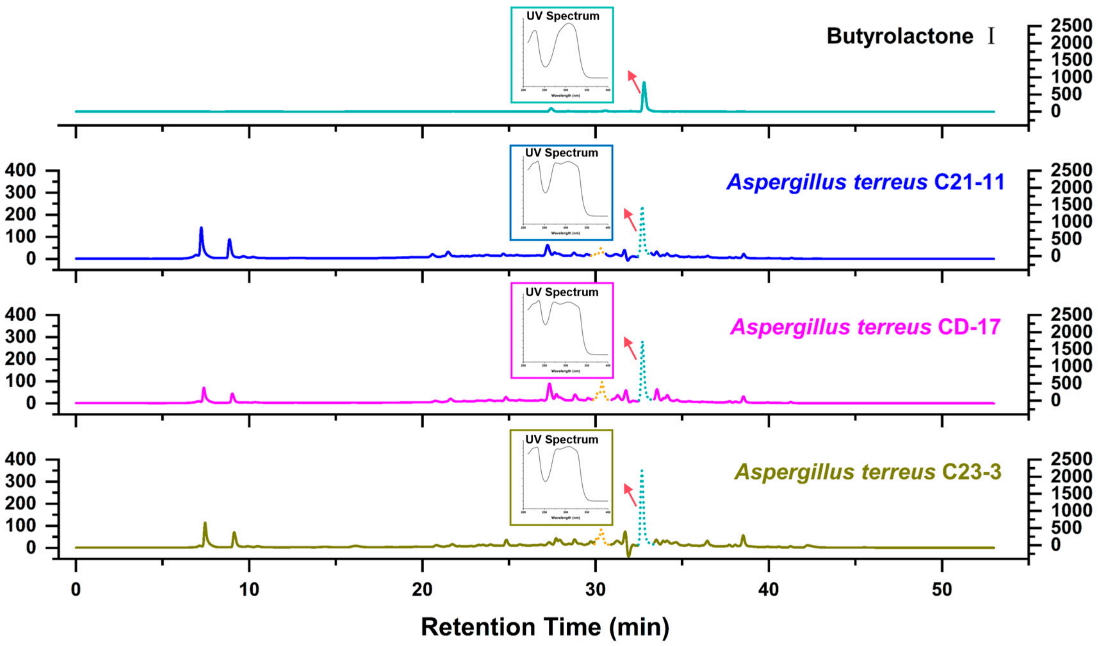

Figure S1. The HPLC analysis of *A. terreus* extract and butylactone I.

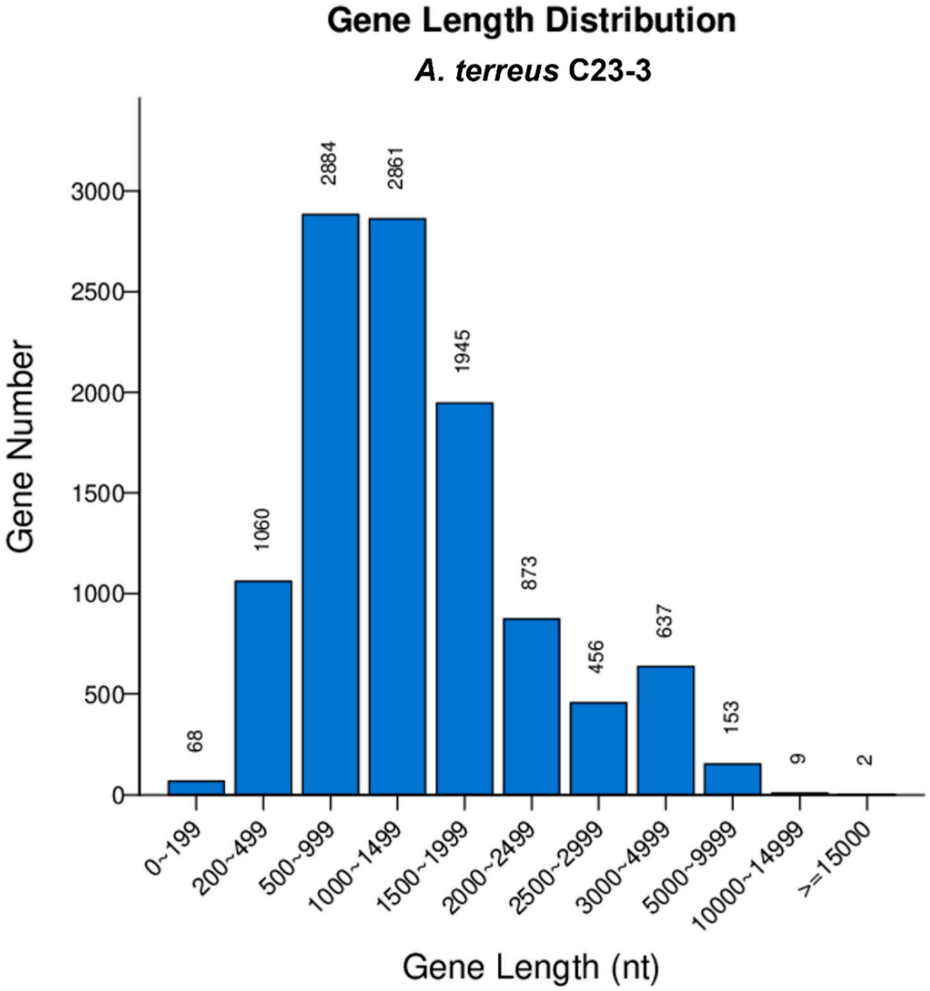

Figure S2. Length of *A. terreus* C23-3 CDS.

Table S3. Statistical assembly of the *A. terreus* C23-3 genome.

Statistics type	Item	Value
Assembly statistics	Total Length (bp)	30,755,296
	N50 Length (bp)	4,133,560
	N90 Length (bp)	2,340,760
	Max Length (bp)	5,258,314
	Min Length (bp)	64,656
	GC Content (%)	52.06
Annotation statistics	Predicted CDS	10,948
	Total CDS Length (bp)	16,144,014
	Average CDS Length (bp)	1,474.61
	CDS Length/Genome Length (%)	52.49
Gene annotation statistics	CAZy	552
	KEGG	4,924
	GO	6,774
	KOG	2,305

Table S4. GNPS and MS-DIAL annotation results.

Compound type	Parent mass	Calculated *m/z*	RT	Adduct	Molecular Formula	GNPS						MS-DIAL				
						**Name**	**Smiles**	**Cosine**	**MZErrorPPM**	**MQScore**	**Bioresource**	**Name**	**Smiles**	**diff(mDa)**	**Bioresource**	**Reported source**
Butyrolactone	425.16	425.1600	4.92	[M+H]+	$C_{24}H_{24}O_7$	Butyrolactone I	CC(=CCC1=C(C=CC(=C1)C[C@@]2(C(=C(C(=O)O2)O)C3=CC=C(C=C3)O)C(=O)OC)O)C	0.87	2	0.873909	*A. terreus*	—	—	—	—	—
	441.16	441.1549	4.02	[M+H]+	$C_{24}H_{24}O_8$	BTL-A	CC(=CCC1=C(C=CC(=C1)CC2(C(=C(C(=O)O2)OC)C3=CC=C(C=C3)O)C(=O)OC)O)C	0.88	13	0.878581	*A. terreus*	—	—	—	—	—
	439.17	439.1757	5.69	[M+H]+	$C_{25}H_{26}O_7$	BTL-B	CC1(C(O1)CC2=C(C=CC(=C2)CC3(C(=C(C(=O)O3)O)C4=CC=C(C=C4)O)C(=O)OC)O)C	0.76	0	0.757135	*A. terreus*	—	—	—	—	—
	357.1017	357.0974	1.857	[M+H]+	$C_{19}H_{16}O_7$	—	—	—	—	—	—	Butyrolactone II	COC(=O)C1(C(=C(C(=O)O1)O)C2=CC=C(C=C2)O)CC3=CC=C(C=C3)O	5.67	*A. terreus*	[45]
Benzodiazepine	416.19	416.1974	4.96	[M+H]+	$C_{25}H_{25}N_3O_3$	Epi-aszonalenin A	CC(=O)N1[C@H]2[C@](C[C@@H]3N2C(=O)C4=CC=CC=C4NC3=O)(C5=CC=CC=C51)C(C)(C)C=	0.78	500	0.776001	*A. terreus*	—	—	—	—	—
	303.10867	303.1109	3.916	[M+Na]+	$C_{17}H_{16}N_2O_2$	—	—	—	—	—	—	Cyclopeptine	CN1C(C(=O)NC2=CC=CC=C2C1=O)CC3=CC=CC=C3	1.71	*Penicillium clavif*	[37]

															orme	
	191.06474	191.0821	6.139	$[M+H]^+$	$C_{10}H_{10}N_2O_2$	—	—	—	—	—	—	4-methyl-2,3,4,5-tetrahydro-1H-1,4-benzodiazepine-2,5-dione	CN1CC(=O)NC2=CC=CC=C2C1=O	5.27	—	—
	229.0371	229.0379	3.672	$[M+K]^+$	$C_{10}H_{10}N_2O_2$	—	—	—	—	—	—	3-methyl-3,4-dihydro-1H-1,4-benzodiazepine-2,5-dione	CC1C(=O)NC2=CC=CC=C2C(=O)N1	0.24	—	—
Statin	405.32	405.2641	7.58	[M+H]+	$C_{24}H_{36}O_5$	Lovastatin	CC[C@H](C)C(=O)O[C@H]1C[C@H](C=C2[C@H]1[C@H]([C@H](C=C2)C)CC[C@@H]3C[C@H](CC(=O)O3)O)C	0.92	1322	0.919831	*A. terreus*	—	—	—	—	—
Meroterpenoid	527.22	527.2281	5.71	[M+H]+	$C_{29}H_{34}O_9$	Territrem B	C[C@@]12CC[C@@]3([C@@]([C@]1(CC4=C(O2)C=C(OC4=O)C5=CC(=C(C(=C5)OC)OC)OC)O)(C(=O)C=CC3(C)C)C)O	0.73	14	0.729557	*A. terreus*	—	—	—	—	—
Steroids	895.554	895.7003	7.76	[2M+Na]+	$C_{27}H_{48}O_4$	Steroids-A	C[C@H](CCCC(C)CO)[C@H]1CC[C@@H]2[C@@]1([C@H](C[C@H]3[C@H]2[C@@H](C[C@H]4[C@@]3(CC[C@H](C4)O)C)O)O)C	0.77	162	0.767369	*A. terreus*	—	—	—	—	—
Benzaldehyde	123.0484	123.0446	9.462	$[M+H]^+$	$C_7H_6O_2$	—	—	—	—	—	—	4-Hydroxyb	C1=CC(=CC=C1C=O)	3.43	*Aspergillus*	[41]

											enzaldehyde			sp	
357.2421	357.2430	8.824	$[M+H]^+$	$C_{23}H_{32}O_3$	—	—	—	—	—	—	Ilicicolin B	CC1=CC(=C(C(=C1C=O)O)C/C=C(\C)/CC/C=C(\C)/CCC=C(C)C)O	0.10	*Fusarium* sp	[39]
390.2646	390.2644	6.655	$[M+NH_4]^+$	$C_{23}H_{32}O_4$	—	—	—	—	—	—	LL-Z1272.Episilon	C[C@@H]1CCC(=O)[C@@H]([C@@]1(C)CC/C(=C/CC2=C(C=C(C(=C2O)C=O)C)O)/C)C	0.55	*Acremonium* sp	[40]
420.193	420.1942	0.637	$[M+NH_4]^+$	$C_{23}H_{27}ClO_4$	—	—	—	—	—	—	BzD-A	CC1C=CC(=O)C(C1(C)/C=C/C(=C/CC2=C(C(=C(C(=C2O)Cl)C)C=O)O)/C)C	0.97	N/A	N/A
406.2599	406.2593	6.635	$[M+NH_4]^+$	$C_{23}H_{32}O_5$	—	—	—	—	—	—	BzD-B	CC1CCC(=O)C(C1(C)CC(/C(=C/CC2=C(C=C(C(=C2O)C=O)C)O)/C)O)C	0.86	*Xylaria* cf. *longipes*	[42]
305.2168	305.4380	5.616	$[M+H]^+$	$C_{19}H_{28}O_3$	—	—	—	—	—	—	Flavoglaucin	CCCCCCCC1=C(C=C(C(=C1C=O)O)CC=C(C)C)O	5.77	*Microsporum*	[43]
197.0801	197.0814	5.194	$[M+H]^+$	$C_{10}H_{12}O_4$	—	—	—	—	—	—	Asaronaldehyde	COC1=CC(=C(C=C1C=O)OC)OC	0.66	*Antrodia cinnamomea*	[44]

BTL represent butyrolactone; **BTL-A** is methyl-2-[[3-[(3,3-dimethyloxiran-2-yl)methyl]-4-hydroxyphenyl]methyl]-4-hydroxy-3-(4-hydroxyphenyl)-5-oxofuran-2-carboxylate; **BTL-B** is methyl-2-[[4-hydroxy-3-(3-methylbut-2-enyl)phenyl]methyl]-3-(4-hydroxyphenyl)-4-methoxy-5-oxofuran-2-carboxylate; **Steroids-A** is 5β-Cholestane-3α,7α,12α,26-tetrol; **BzD** represent benzaldehyde derivatives; **BzD-A** is 5-chloro-2,4-dihydroxy-6-methyl-3-[(2E,4E)-3-methyl-5-(1,2,6-trimethyl-5-oxocyclohex-3-en-1-yl)penta-2,4-

dienyl]benzaldehyde; **BzD-B** is 2,4-dihydroxy-3-[(E)-4-hydroxy-3-methyl-5-(1,2,6-trimethyl-3-oxocyclohexyl)pent-2-enyl]-6-methylbenzaldehyde.

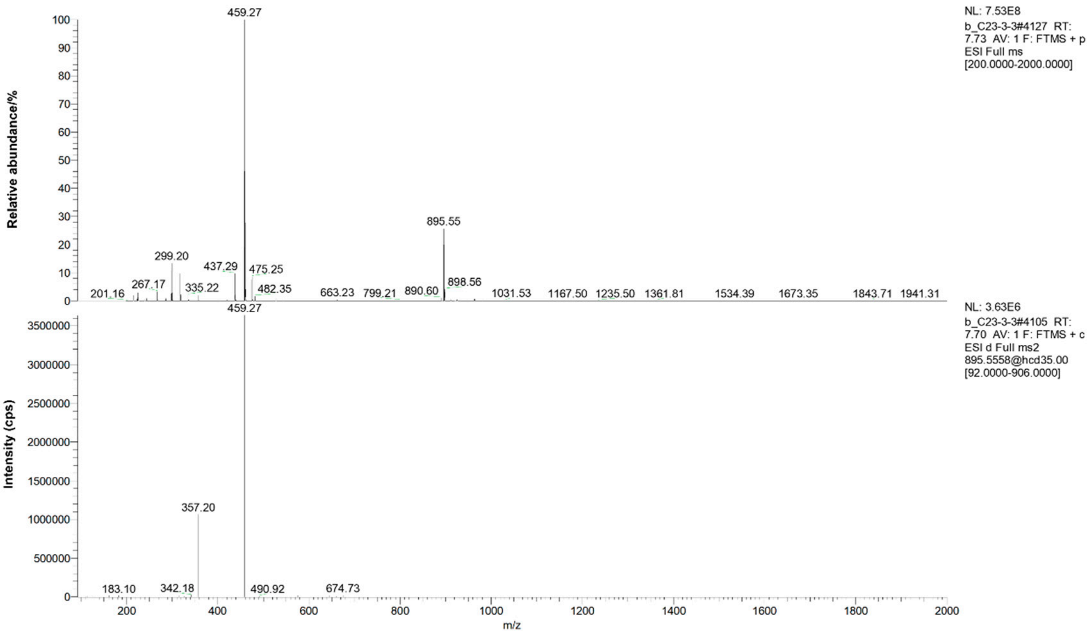

Figure S3. MS and MS^2 spectrogram of peak 7.

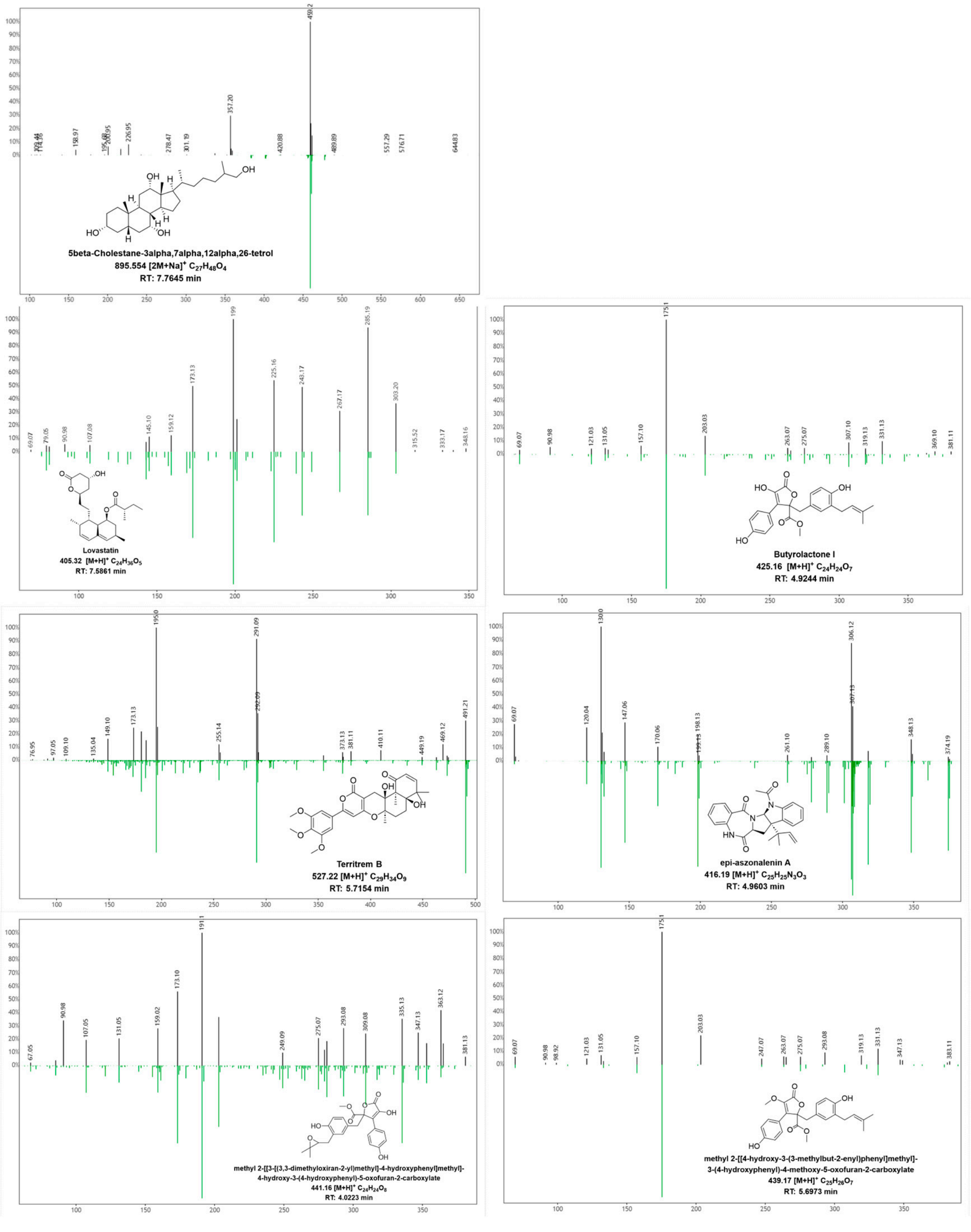

Figure S4. MS^2 mirror matching spectra of GNPS annotation.

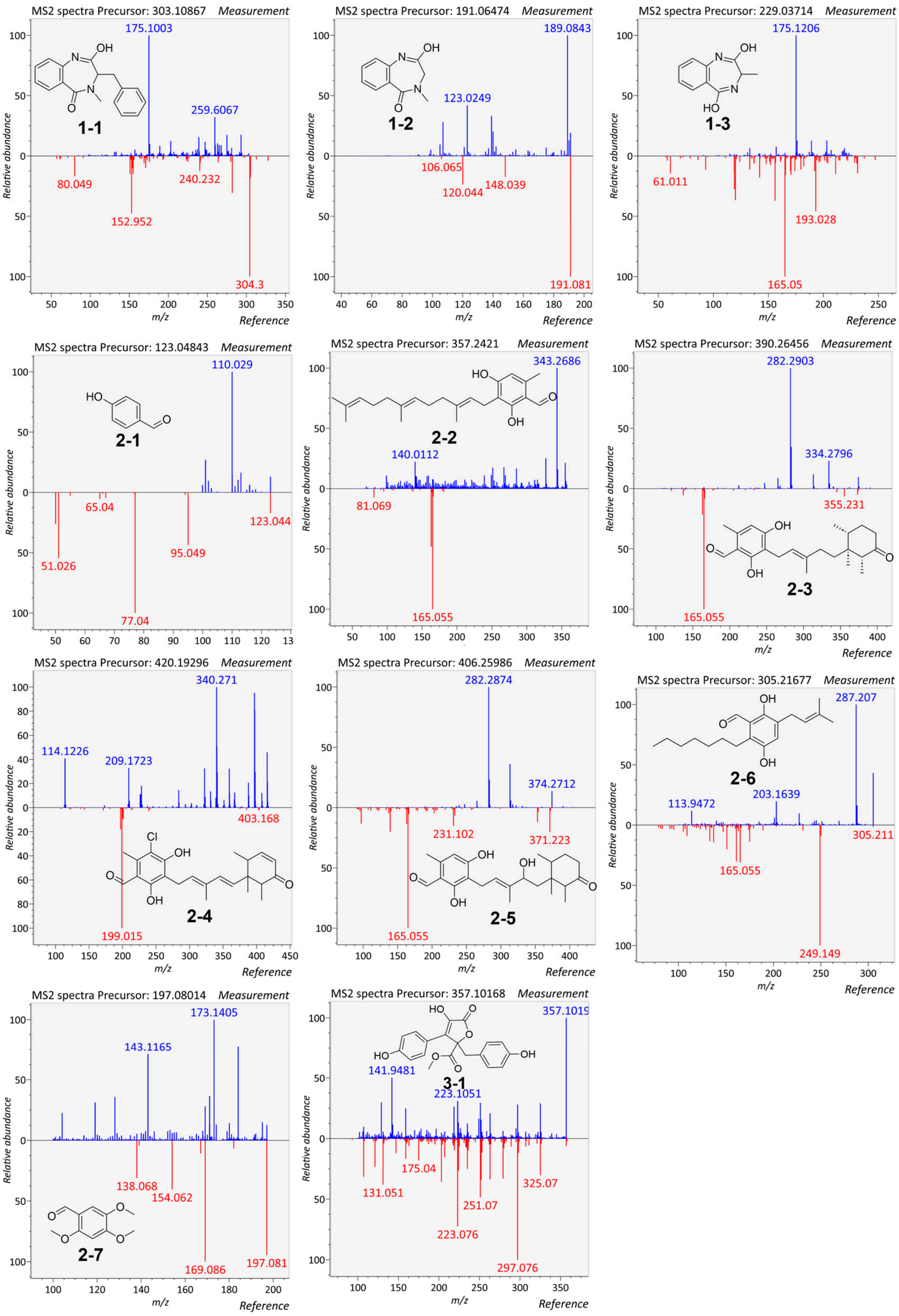

Figure S5. MS^2 mirror matching spectra of MS-DIAL annotation.

Table S5. Result docking with AChE.

Ligand	Binding energy (kcal/mol)	Interacting amino acids			
		Hydrogen bond	Hydrophobic	Electrostatic	π-π stacking
2,4-dihydroxy-5,6-dimethyl benzaldehyde	-6.9	Tyr124, Ser125, Gly126	Trp86, His447	-	Trp86
Aspulvinone B1	-12.4	Ser203, Phe295, Arg296	Trp86, Trp286	-	Tyr124, Phe338, Tyr341
Aspulvinone H	-11.9	Gly122, Tyr124, Ser293	Tyr72, Trp86, Trp286	-	Tyr124, Phe338, Tyr341, His447
Asterrelenin	-10.4	Asp74, Tyr124	Trp86, Tyr337, His447	Asp74	-
Epi-aszonalenin A	-8.5	Tyr341	Trp286, Leu289, Val294	Trp286	-
Butyrolactone I	-10.3	Asn87	Tyr72, Trp86, Trp286, Tyr337	-	Tyr124, Tyr341
Butyrolactone III	-10.3	Trp86, Tyr124, Tyr337	Tyr72, Trp86, Trp286, Tyr341, His447	-	Tyr124, Tyr341
BTL-A	-9.6	Trp286, Ser293, Phe295	Leu289, Phe338, Tyr341	-	Tyr72
BTL-B	-8.5	Tyr72, Arg296	Tyr72, Trp286, His287, Leu289	-	Trp286
Lovastatin	-11.2	Ser125	Trp86, Tyr124, Leu130, Tyr133, Tyr337, Phe338, His447	-	-
Territrem B	-10.5	-	Trp86, Tyr124, Phe297	-	Trp286, Tyr341, Phe338
P_AChE	-11.7	Phe295	Trp286, Tyr337, Phe338, Tyr341	-	Trp86, Trp286, Tyr341
P_CDK5/p25	-11.1	-	-	-	Trp86, Trp286, Tyr337, Tyr341, His447
P_GSK-3β	-11.3	Phe295	Trp86	-	Trp286, Tyr337, Tyr341
P_MAO-B	-11.7	Tyr72	Tyr337	-	Trp86, Trp286, Tyr337, Tyr341

BTL-A, methyl 2-[[3-[(3,3-dimethyloxiran-2-yl)methyl]-4-hydroxyphenyl]methyl]-4-hydroxy-3-(4-hydroxyphenyl)-5-oxofuran-2-carboxylate; **BTL-B**, methyl 2-[[4-hydroxy-3-(3-methylbut-2-enyl)phenyl]methyl]-3-(4-hydroxyphenyl)-4-methoxy-5-oxofuran-2-carboxylate; **P_AChE**, positive control of AChE; **P_CDK5/p25**, positive control of CDK5/p25; **P_GSK-3β**, positive control of GSK-3β; **P_MAO-B**, positive control of MAO-B.

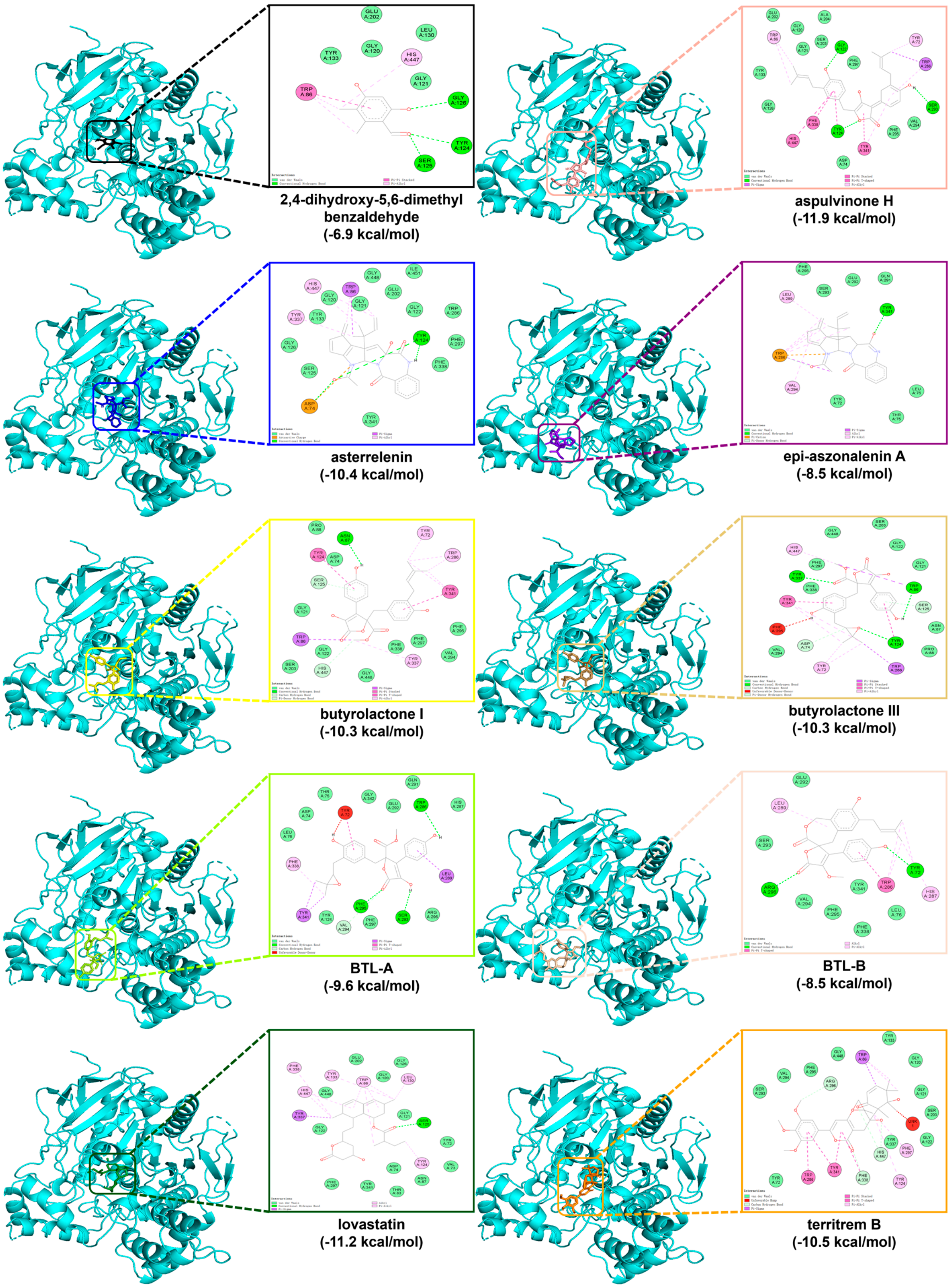

Figure S6. Target compounds docking with AChE (except aspulvinone B1). BTL-A is methyl 2-[[3-[(3,3-dimethyloxiran-2-yl)methyl]-4-hydroxyphenyl]methyl]-4-hydroxy-3-(4-hydroxyphenyl)-5-oxofuran-2-carboxylate; BTL-B is methyl 2-[[4-hydroxy-3-(3-methylbut-2-enyl)phenyl]methyl]-3-(4-hydroxyphenyl)-4-methoxy-5-oxofuran-2-carboxylate.

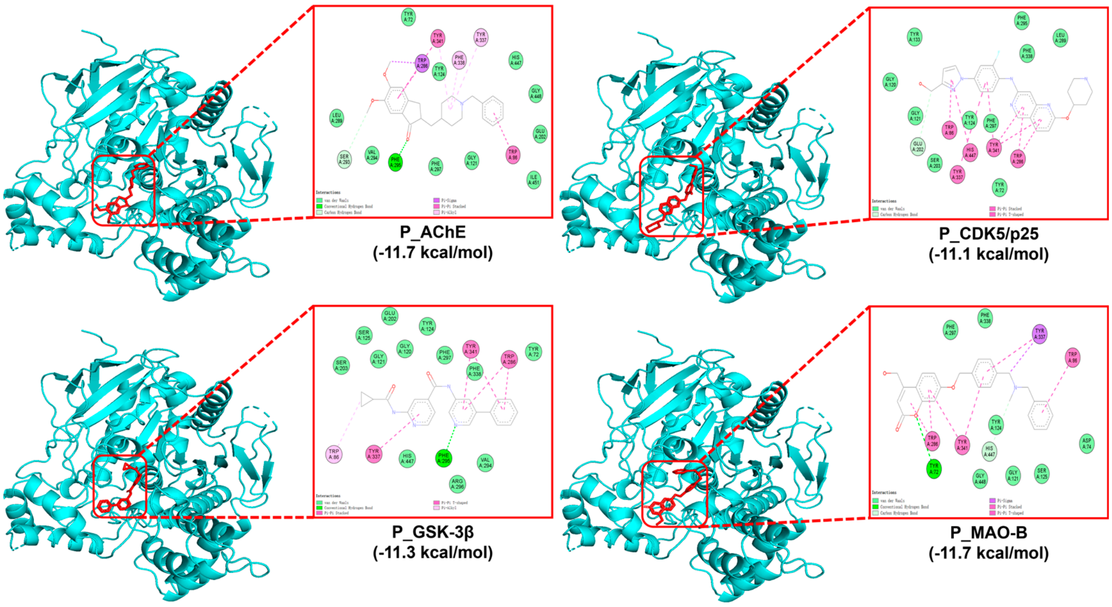

Figure S7. Positive control docking with AChE. P_AChE, positive control of AChE; P_CDK5/p25, positive control of CDK5/p25; P_GSK-3β, positive control of GSK-3β; P_MAO-B, positive control of MAO-B.

Table S6. Result docking with CDK5/p25.

Ligand	Binding energy (kcal/mol)	Interacting amino acids			
		Hydrogen bond	Hydrophobic	Electrostatic	π-π stacking
2,4-dihydroxy-5,6-dimethyl benzaldehyde	-6.0	Cys83, Asp86	Ile10, Val18, Leu133	-	-
Aspulvinone B1	-9.9	Glu81, Cys83	Ile10, Val18, Ala31, Lys33, Val64, Phe80, Cys83, Leu133, Ala143	Asp144	-
Aspulvinone H	-9.7	Cys83	Ile10, Val18, Lys20, Ala31, Val64, Phe80, Phe82, Lys89, Leu133, Ala143	-	-
Asterrelenin	-10.2	Gln130, Asp144	Val18, Ala31, Lys33, Val64, Phe80, Leu133	Asp144	-
Epi-aszonalenin A	-6.8	-	Lys33, Val35, Val44, Ala48, Leu147	Asp144	-
Butyrolactone I	-9.2	Glu81, Cys83	Ile10, Val18, Ala31, Lys33, Val64, Phe80, Leu133, Ala143	Asp86	-
Butyrolactone III	-8.5	Glu12, Asp86	Ile10, Val18, Ala31, Lys33, Phe80, Leu133, Ala143	Asp86	-
BTL-A	-8.1	Glu12	Ile10, Val18, Lys33, Val64, Phe80, Leu133, Ala143	-	-
BTL-B	-10.1	Lys33, Glu81	Ile10, Val18, Ala31, Phe80, Phe82, Leu133	-	-
Lovastatin	-8.9	Asp144	Ile10, Val18, Ala31, Val64, Phe80, Cys83, Leu133, Ala143	-	-
Territrem B	-9.0	Asp144	Ile10, Val18, Val64, Phe80, Lys89, Leu133, Ala143	Asp86	-
P_AChE	-9.7	-	Ile10, Val18, Ala31, Val64, Phe80, Cys83, Lys89, Leu133, Ala143	-	Phe80
P_CDK5/p25	-8.9	Cys83, Asp86, Lys89	Ile10, Val18, Ala31, Cys83, Leu133	-	-
P_GSK-3β	-9.1	Asp84	Ile10, Val18, Ala31, Val64, Cys83, Lys89, Leu133, Ala143	-	Phe80, Gln85
P_MAO-B	-8.9	Asp144	Ile10, Val18, Leu133, Ala143	Glu8	Gln85

BTL-A, methyl 2-[[3-[(3,3-dimethyloxiran-2-yl)methyl]-4-hydroxyphenyl]methyl]-4-hydroxy-3-(4-hydroxyphenyl)-5-oxofuran-2-carboxylate; **BTL-B**, methyl 2-[[4-hydroxy-3-(3-methylbut-2-enyl)phenyl]methyl]-3-(4-hydroxyphenyl)-4-methoxy-5-oxofuran-2-carboxylate; **P_AChE**, positive control of AChE; **P_CDK5/p25**, positive control of CDK5/p25; **P_GSK-3β**, positive control of GSK-3β; **P_MAO-B**, positive control of MAO-B.

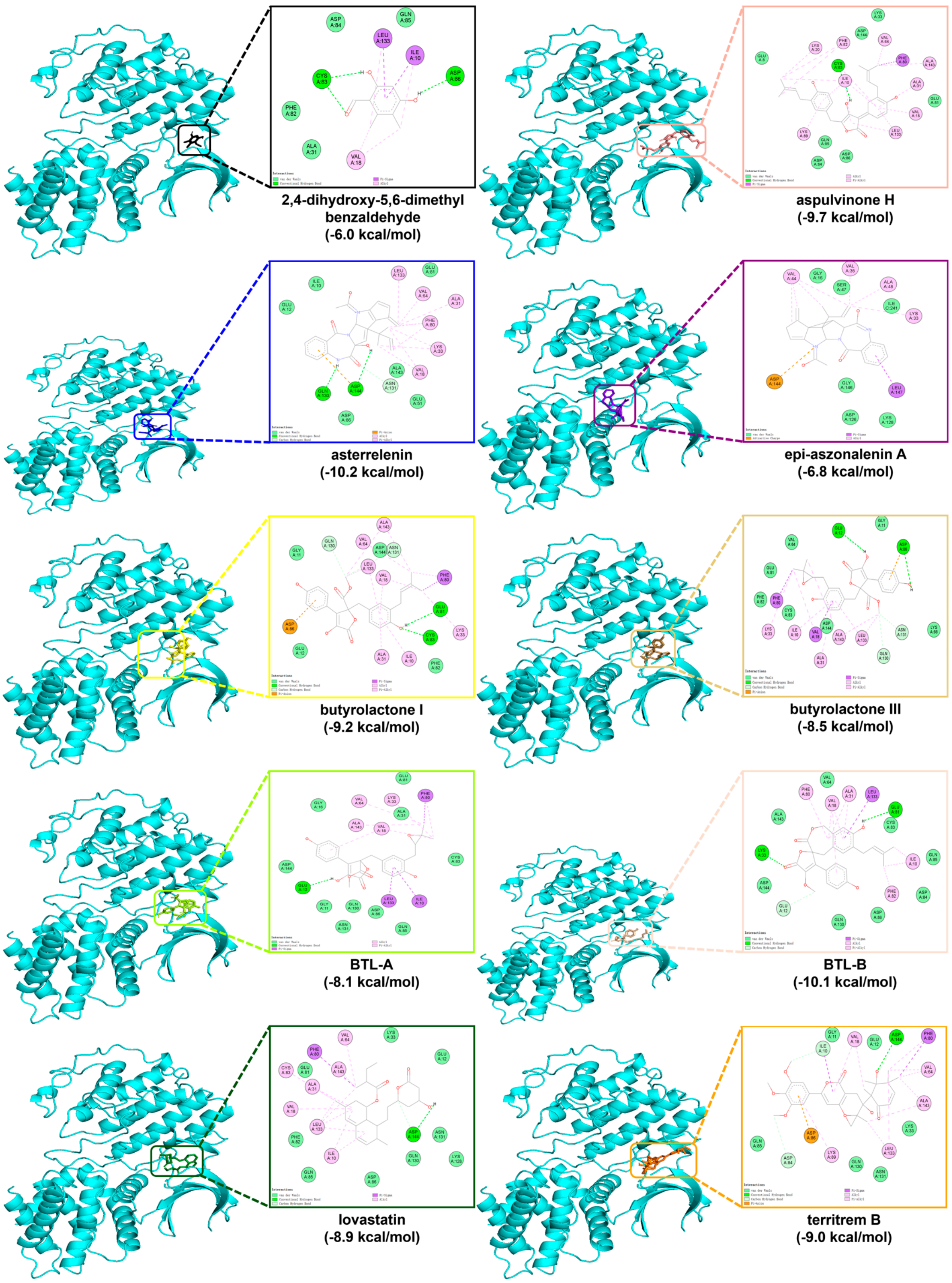

Figure S8. Target compounds docking with CDK5/p25 (except aspulvinone B1). BTL-A is methyl 2-[[3-[(3,3-dimethyloxiran-2-yl)methyl]-4-hydroxyphenyl]methyl]-4-hydroxy-3-(4-hydroxyphenyl)-5-oxofuran-2-carboxylate; BTL-B is methyl 2-[[4-hydroxy-3-(3-methylbut-2-enyl)phenyl]methyl]-3-(4-hydroxyphenyl)-4-methoxy-5-oxofuran-2-carboxylate.

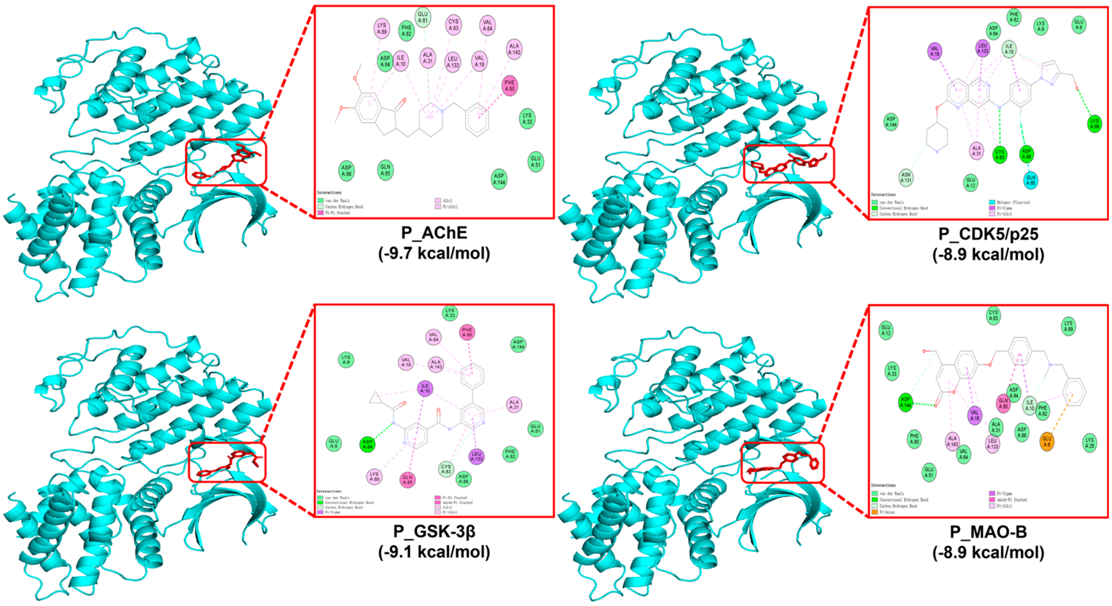

Figure S9. Positive control docking with CDK5/p25. P_AChE, positive control of AChE; P_CDK5/p25, positive control of CDK5/p25; P_GSK-3β, positive control of GSK-3β; P_MAO-B, positive control of MAO-B.

Table S7. Result docking with GSK-3β.

Ligand	Binding energy (kcal/mol)	Interacting amino acids			
		Hydrogen bond	Hydrophobic	Electrostatic	π-π stacking
2,4-dihydroxy-5,6-dimethyl benzaldehyde	-5.5	Asp133	Ala83, Val110, Leu132, Leu188, Cys199	-	-
Aspulvinone B1	-9.5	-	Phe67, Val70, Ala83, Lys85, Leu132, Tyr140, Arg141, Leu188, Cys199	Arg141	Tyr134
Aspulvinone H	-8.9	-	Phe67, Val70, Ala83, Lys85, Leu132, Leu188, Cys199	-	-
Asterrelenin	-10.7	Gln185	Ile62, Phe67, Val70, Ala83, Lys85, Val110, Leu132, Leu188, Cys199	Asp200	-
Epi-aszonalenin A	-10.1	Ile62, Gln185	Phe67, Val70, Ala83, Lys85, Val110, Leu132, Leu188, Cys199	-	-
Butyrolactone I	-8.4	Lys85, Cys199, Asp200	Val70, Ala83, Leu188	-	Phe67
Butyrolactone III	-8.6	Lys85, Cys199, Asp200	Ile62, Val70, Ala83, Leu188	-	Phe67
BTL-A	-8.3	Val135	Ile62, Val70, Ala83, Lys85, Val110, Leu132, Leu188, Cys199	-	Tyr134
BTL-B	-8.7	-	Val70, Ala83, Lys85, Leu132, Leu188, Cys199	Cys199	-
Lovastatin	-8.4	Arg141	Phe67, Val70, Ala83, Lys85, Tyr134, Leu188	-	-
Territrem B	-8.5	Ser66, Lys183	Ile62, Phe67, Val70, Cys199	Asp181, Asp200	-
P_AChE	-7.9	-	Val70, Lys85, Leu132	Cys199	-
P_CDK5/p25	-8.9	Lys85, Arg141, Lys183	Val70, Ala83, Leu132, Leu188, Cys199	Lys183, Cys199, Asp200	-
P_GSK-3β	-8.6	Asp200	Phe67, Val70, Ala83, Lys85, Leu188, Cys199	Cys199	Phe67
P_MAO-B	-7.8	Thr138, Arg141	Val70, Ala83, Leu188	Cys199, Asp200	-

BTL-A , methyl 2-[[3-[(3,3-dimethyloxiran-2-yl)methyl]-4-hydroxyphenyl]methyl]-4-hydroxy-3-(4-hydroxyphenyl)-5-oxofuran-2-carboxylate; **BTL-B** , methyl 2-[[4-hydroxy-3-(3-methylbut-2-enyl)phenyl]methyl]-3-(4-hydroxyphenyl)-4-methoxy-5-oxofuran-2-carboxylate; **P_AChE**, positive control of AChE; **P_CDK5/p25**, positive control of CDK5/p25; **P_GSK-3β**, positive control of GSK-3β; **P_MAO-B**, positive control of MAO-B.

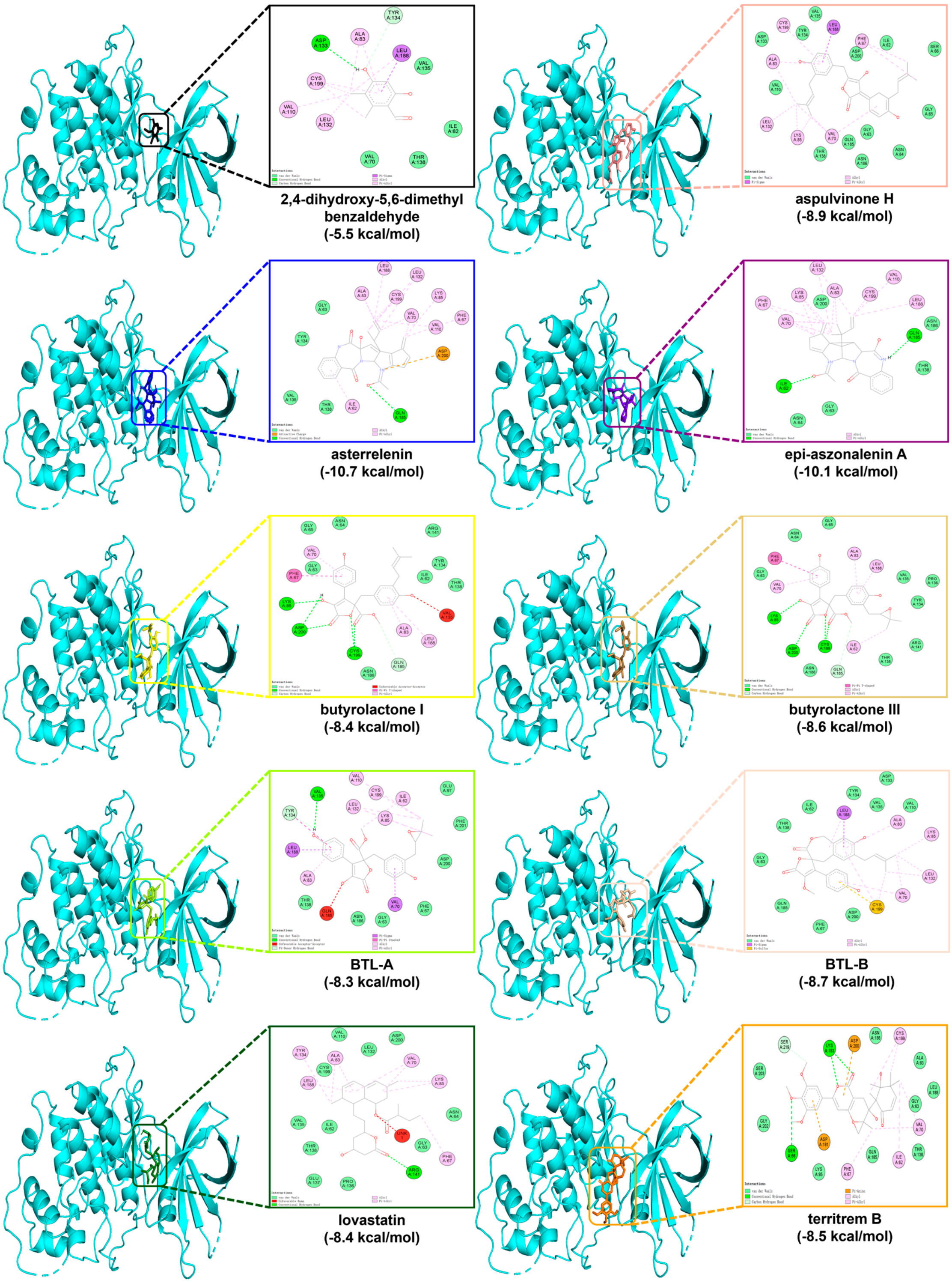

Figure S10. Target compounds docking with GSK-3β (except aspulvinone B1). BTL-A is methyl 2-[[3-[(3,3-dimethyloxiran-2-yl)methyl]-4-hydroxyphenyl]methyl]-4-hydroxy-3-(4-hydroxyphenyl)-5-oxofuran-2-carboxylate; BTL-B is methyl 2-[[4-hydroxy-3-(3-methylbut-2-enyl)phenyl]methyl]-3-(4-hydroxyphenyl)-4-methoxy-5-oxofuran-2-carboxylate.

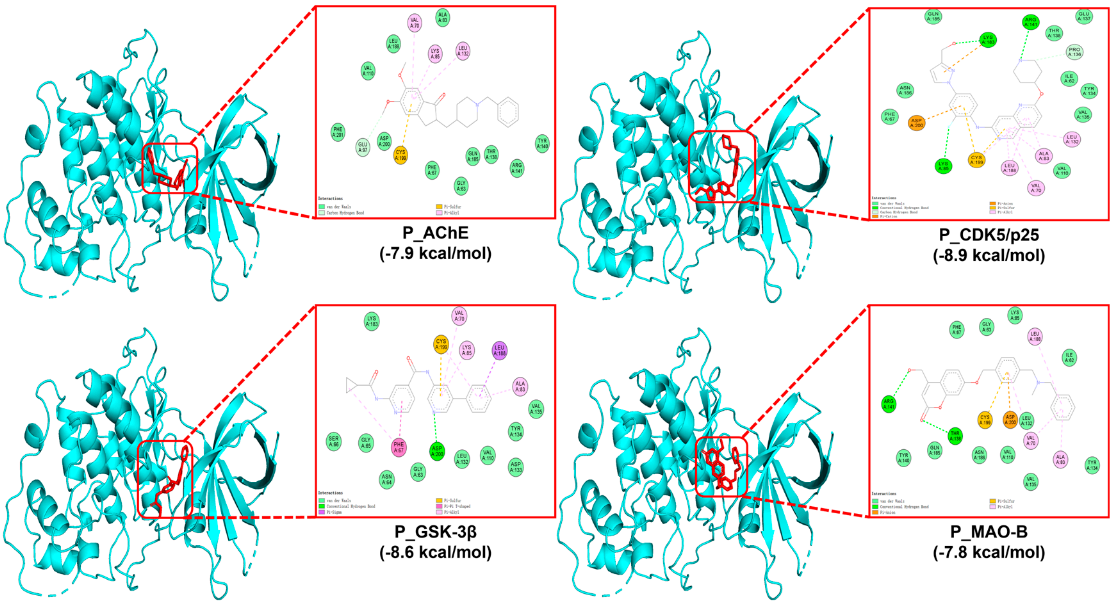

Figure S11. Positive control docking with GSK-3β. P_AChE, positive control of AChE; P_CDK5/p25, positive control of CDK5/p25; P_GSK-3β, positive control of GSK-3β; P_MAO-B, positive control of MAO-B.

Table S8. Result docking with MAO-B.

Ligand	Binding energy (kcal/mol)	Interacting amino acids			
		Hydrogen bond	Hydrophobic	Electrostatic	π-π stacking
2,4-dihydroxy-5,6-dimethyl benzaldehyde	-7.2	Ser59, Tyr60, Gln206, Met436	Tyr398, Tyr435	-	Tyr435
Aspulvinone B1	-13.4	Gln206, Tyr398	Leu171, Ile199, Leu164, Tyr398, Tyr435	Cys172	Tyr326, Tyr398
Aspulvinone H	-12.2	Pro102, Cys172	His115, Phe118,	Cys172	Tyr326, Tyr398
Asterrelenin	-6.2	Glu84, Thr202	Pro102	Glu84	-
Epi-aszonalenin A	-6.0	-	Arg100, Pro102	Glu84	-
Butyrolactone I	-8.9	Ser59, Tyr60, Lys296, Met436	Gly57, Leu171, Tyr326, Phe343, Tyr398	Met436	Tyr398
Butyrolactone III	-8.8	Gly58, Ser59, Lsy296	Arg42, Tyr435, Ala439	Cys397	Tyr398
BTL-A	-9.6	Ser59, Lys296	Arg42, Lys296, Phe343, Cys397, Tyr398	-	Tyr398
BTL-B	-5.8	Asn203	Pro102, Thr202	Glu84	-
Lovastatin	-8.9	Lys296	Tyr60, Val294, Phe343, Trp388, Cys397, Tyr398, Tyr435, Met436	-	-
Territrem B	-6.0	Asn203	Gly101, Pro102	-	-
P_AChE	-11.1	Ser59	Leu171, Cys172, Ile199, Ile316, Tyr326	-	Tyr398, Tyr435
P_CDK5/p25	-11.8	Met436	Arg42	Cys397	Leu171, Tyr398
P_GSK-3β	-11.4	Cys172, Tyr435	Leu171, Ile199, Phe343, Tyr398	Cys172	Phe103, Phe118, Phe168, Tyr326
P_MAO-B	-12.4	-	Leu171, Ile199, Ile316	Cys172	Phe103, Phe118, Trp119, Phe168, Tyr326, Tyr398

BTL-A , methyl 2-[[3-[(3,3-dimethyloxiran-2-yl)methyl]-4-hydroxyphenyl]methyl]-4-hydroxy-3-(4-hydroxyphenyl)-5-oxofuran-2-carboxylate; **BTL-B** , methyl 2-[[4-hydroxy-3-(3-methylbut-2-enyl)phenyl]methyl]-3-(4-hydroxyphenyl)-4-methoxy-5-oxofuran-2-carboxylate; **P_AChE**, positive control of AChE; **P_CDK5/p25**, positive control of CDK5/p25; **P_GSK-3β**, positive control of GSK-3β; **P_MAO-B**, positive control of MAO-B.

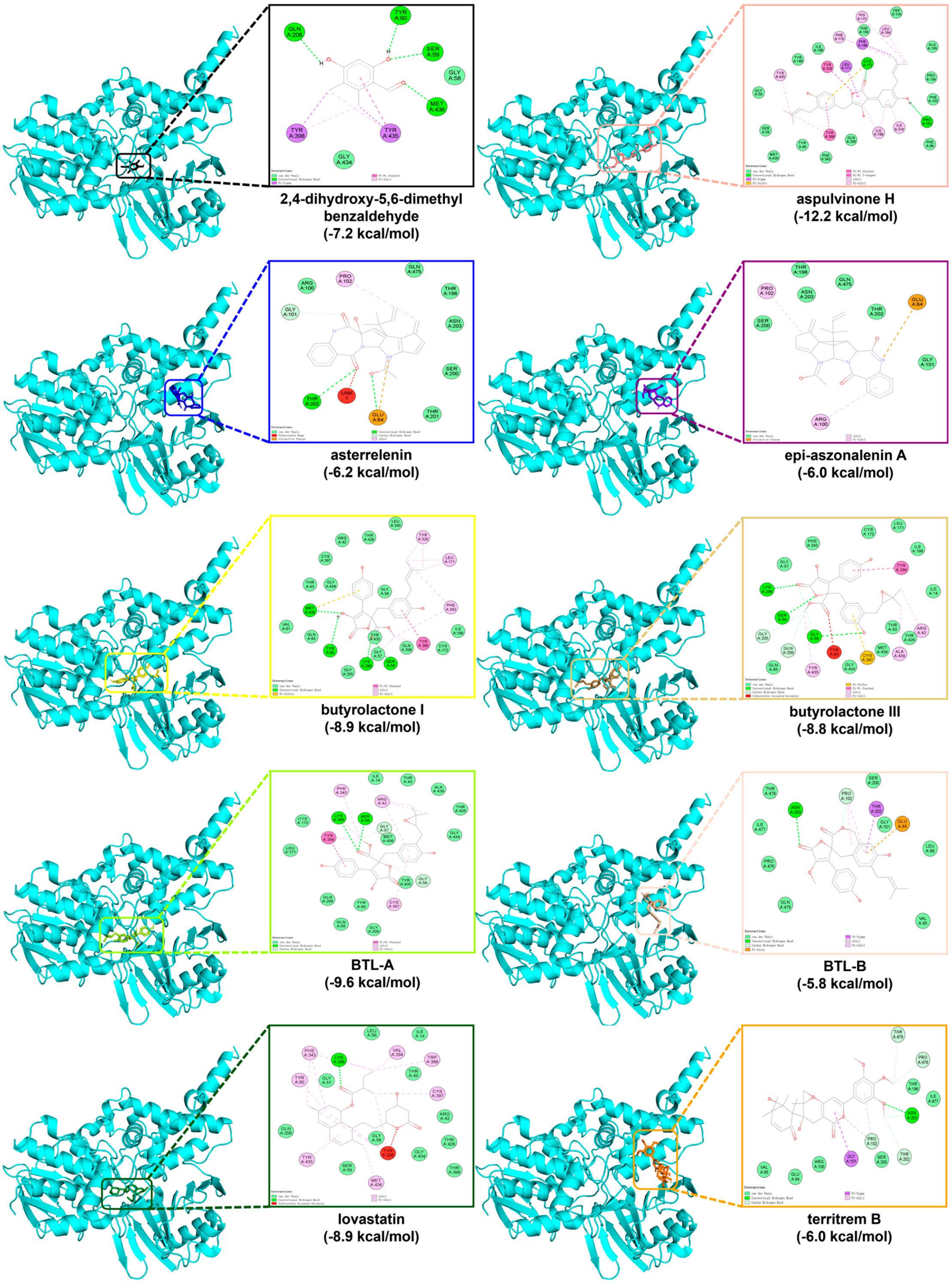

Figure S12. Target compounds docking with MAO-B (except aspulvinone B1). BTL-A is methyl 2-[[3-[(3,3-dimethyloxiran-2-yl)methyl]-4-hydroxyphenyl]methyl]-4-hydroxy-3-(4-hydroxyphenyl)-5-oxofuran-2-carboxylate; BTL-B is methyl 2-[[4-hydroxy-3-(3-methylbut-2-enyl)phenyl]methyl]-3-(4-hydroxyphenyl)-4-methoxy-5-oxofuran-2-carboxylate.

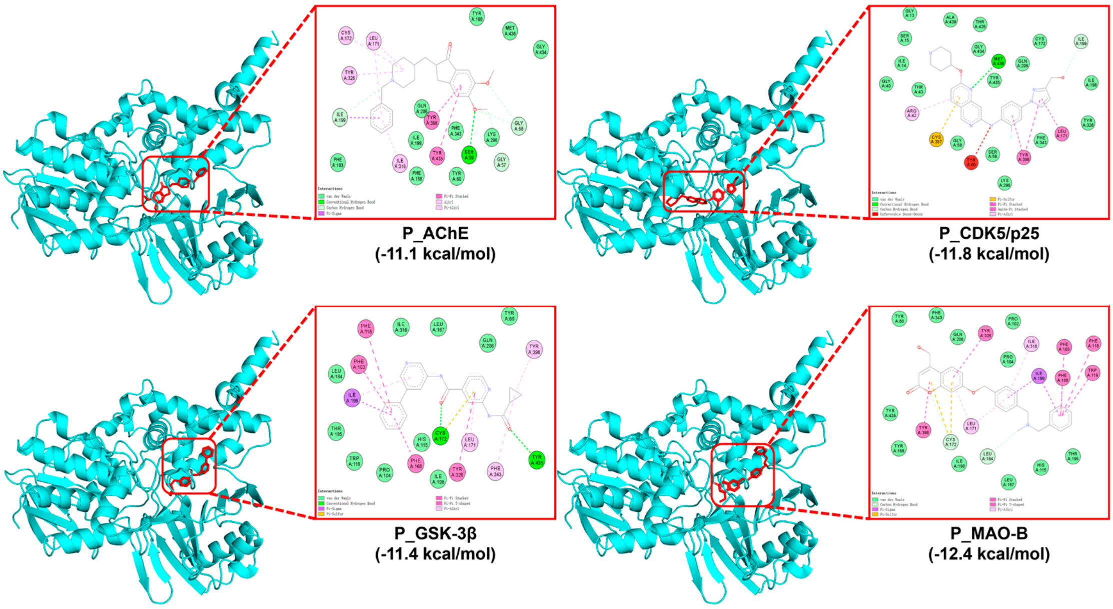

Figure S13. Positive control docking with MAO-B. P_AChE, positive control of AChE; P_CDK5/p25, positive control of CDK5/p25; P_GSK-3β, positive control of GSK-3β; P_MAO-B, positive control of MAO-B.

Table S9. ADMET and drug-likeness properties of 2,4-dihydroxy-5,6-dimethyl benzaldehyde through the online prediction tool ADMETlab 3.0.

Property	Value
Absorption	
Caco-2 permeability	-5.02
Madin–Darby canine kidney cells (MDCK) permeability	-4.758
P-glycoprotein (Pgp)-inhibitor	0.233
P-glycoprotein (Pgp)-substrate	0.152
Human intestinal absorption (HIA)	0.004
20% bioavailability (F20%)	0.232
Distribution	
Plasma protein binding (PPB)	86.4%
Volume distribution (VD)	-0.226
Blood–brain barrier (BBB) penetration	0.673
The fraction unbound in plasma (Fu)	11.6%
Metabolism	
CYP1A2-inhibitor	1.0
CYP1A2-substrate	0.954
CYP2C19-inhibitor	0.452
CYP2C19-substrate	0.655
CYP2C9-inhibitor	0.03
CYP2C9-substrate	1.0
CYP2D6-inhibitor	0.105
CYP2D6-substrate	0.992
CYP3A4-inhibitor	0.04
CYP3A4-substrate	0.28
Excretion	
Clearance	1.986
The half-life ($T_{1/2}$)	1.996
Toxicity	
hERG blockers	0.037
Human hepatotoxicity (H-HT)	0.362
Drug-induced liver injury (DILI)	0.185
Ames toxicity	0.468
Rat oral acute toxicity	0.371
Maximum recommended daily dose (FDAMDD)	0.363
Skin sensitization	0.939
Carcinogenicity	0.475
Eye corrosion	0.990
Eye irritation	0.999
Respiratory toxicity	0.927
Drug-likeness	
MCE-18	8.0
Lipinski rule	0.0

Table S9 cont.

Property	Value
Pfzer rule	0.0
Golden triangle	1.0
GSK rule	0.0

Table S10. ADMET and drug-likeness properties of aspulvinone B1 through the online prediction tool ADMETlab 3.0.

Property	Value
Absorption	
Caco-2 permeability	-5.03
Madin–Darby canine kidney cells (MDCK) permeability	-4.727
P-glycoprotein (Pgp)-inhibitor	0.068
P-glycoprotein (Pgp)-substrate	0.0
Human intestinal absorption (HIA)	0.239
20% bioavailability (F20%)	1.0
Distribution	
Plasma protein binding (PPB)	96.6%
Volume distribution (VD)	0.043
Blood–brain barrier (BBB) penetration	0.0
The fraction unbound in plasma (Fu)	2.6%
Metabolism	
CYP1A2-inhibitor	0.959
CYP1A2-substrate	0.0
CYP2C19-inhibitor	1.0
CYP2C19-substrate	0.098
CYP2C9-inhibitor	0.999
CYP2C9-substrate	0.698
CYP2D6-inhibitor	0.012
CYP2D6-substrate	0.47
CYP3A4-inhibitor	0.001
CYP3A4-substrate	0.0
Excretion	
Clearance	2.12
The half-life ($T_{1/2}$)	1.561
Toxicity	
hERG blockers	0.058
Human hepatotoxicity (H-HT)	0.701
Drug-induced liver injury (DILI)	0.911
Ames toxicity	0.457
Rat oral acute toxicity	0.541
Maximum recommended daily dose (FDAMDD)	0.511
Skin sensitization	0.995
Carcinogenicity	0.227
Eye corrosion	0.000
Eye irritation	0.788
Respiratory toxicity	0.915
Drug-likeness	
MCE-18	55.636
Lipinski rule	0.0

Table S10 cont.

Property	Value
Pfzer rule	0.0
Golden triangle	0.0
GSK rule	1.0

Table S11. ADMET and drug-likeness properties of aspulvinone H through the online prediction tool ADMETlab 3.0.

Property	Value
Absorption	
Caco-2 permeability	-5.129
Madin–Darby canine kidney cells (MDCK) permeability	-4.791
P-glycoprotein (Pgp)-inhibitor	0.005
P-glycoprotein (Pgp)-substrate	0.0
Human intestinal absorption (HIA)	0.782
20% bioavailability (F20%)	1.0
Distribution	
Plasma protein binding (PPB)	94.0%
Volume distribution (VD)	1.296
Blood–brain barrier (BBB) penetration	0.0
The fraction unbound in plasma (Fu)	5.4%
Metabolism	
CYP1A2-inhibitor	0.767
CYP1A2-substrate	0.0
CYP2C19-inhibitor	1.0
CYP2C19-substrate	0.962
CYP2C9-inhibitor	1.0
CYP2C9-substrate	0.068
CYP2D6-inhibitor	0.016
CYP2D6-substrate	0.998
CYP3A4-inhibitor	0.008
CYP3A4-substrate	0.0
Excretion	
Clearance	4.338
The half-life ($T_{1/2}$)	1.131
Toxicity	
hERG blockers	0.014
Human hepatotoxicity (H-HT)	0.739
Drug-induced liver injury (DILI)	0.992
Ames toxicity	0.285
Rat oral acute toxicity	0.4
Maximum recommended daily dose (FDAMDD)	0.167
Skin sensitization	1.0
Carcinogenicity	0.063
Eye corrosion	0.006
Eye irritation	0.923
Respiratory toxicity	0.659
Drug-likeness	
MCE-18	42.0
Lipinski rule	0.0

Table S11 cont.

Property	Value
Pfzer rule	0.0
Golden triangle	0.0
GSK rule	1.0

Table S12. ADMET and drug-likeness properties of asterrelenin through the online prediction tool ADMETlab 3.0.

Property	Value
Absorption	
Caco-2 permeability	-5.386
Madin–Darby canine kidney cells (MDCK) permeability	-4.968
P-glycoprotein (Pgp)-inhibitor	0.767
P-glycoprotein (Pgp)-substrate	0.999
Human intestinal absorption (HIA)	0.005
20% bioavailability (F20%)	0.963
Distribution	
Plasma protein binding (PPB)	93.8%
Volume distribution (VD)	1.125
Blood–brain barrier (BBB) penetration	0.003
The fraction unbound in plasma (Fu)	5.9%
Metabolism	
CYP1A2-inhibitor	0.0
CYP1A2-substrate	0.8
CYP2C19-inhibitor	0.028
CYP2C19-substrate	1.0
CYP2C9-inhibitor	0.003
CYP2C9-substrate	0.0
CYP2D6-inhibitor	0.0
CYP2D6-substrate	0.0
CYP3A4-inhibitor	0.006
CYP3A4-substrate	0.989
Excretion	
Clearance	3.478
The half-life ($T_{1/2}$)	0.647
Toxicity	
hERG blockers	0.045
Human hepatotoxicity (H-HT)	0.909
Drug-induced liver injury (DILI)	0.962
Ames toxicity	0.483
Rat oral acute toxicity	0.212
Maximum recommended daily dose (FDAMDD)	0.513
Skin sensitization	0.990
Carcinogenicity	0.442
Eye corrosion	0.0
Eye irritation	0.62
Respiratory toxicity	0.529
Drug-likeness	
MCE-18	110.242
Lipinski rule	0.0

Table S12 cont.

Property	Value
Pfzer rule	0.0
Golden triangle	0.0
GSK rule	1.0

Table S13. ADMET and drug-likeness properties of epi-aszonalenin A through the online prediction tool ADMETlab 3.0.

Property	Value
Absorption	
Caco-2 permeability	-4.903
Madin–Darby canine kidney cells (MDCK) permeability	-4.746
P-glycoprotein (Pgp)-inhibitor	0.44
P-glycoprotein (Pgp)-substrate	0.891
Human intestinal absorption (HIA)	0.0
20% bioavailability (F20%)	0.008
Distribution	
Plasma protein binding (PPB)	92.2%
Volume distribution (VD)	1.376
Blood–brain barrier (BBB) penetration	0.166
The fraction unbound in plasma (Fu)	5.6%
Metabolism	
CYP1A2-inhibitor	0.02
CYP1A2-substrate	0.091
CYP2C19-inhibitor	0.845
CYP2C19-substrate	0.988
CYP2C9-inhibitor	0.056
CYP2C9-substrate	0.0
CYP2D6-inhibitor	0.0
CYP2D6-substrate	0.0
CYP3A4-inhibitor	0.826
CYP3A4-substrate	0.999
Excretion	
Clearance	5.209
The half-life ($T_{1/2}$)	0.398
Toxicity	
hERG blockers	0.036
Human hepatotoxicity (H-HT)	0.971
Drug-induced liver injury (DILI)	0.997
Ames toxicity	0.179
Rat oral acute toxicity	0.186
Maximum recommended daily dose (FDAMDD)	
Skin sensitization	0.988
Carcinogenicity	0.121
Eye corrosion	0.0
Eye irritation	0.658
Respiratory toxicity	0.466
Drug-likeness	
MCE-18	103.758
Lipinski rule	0.0

Table S13 cont.

Property	Value
Pfzer rule	0.0
Golden triangle	0.0
GSK rule	1.0

Table S14. ADMET and drug-likeness properties of butyrolactone III through the online prediction tool ADMETlab 3.0.

Property	Value
Absorption	
Caco-2 permeability	-5.131
Madin–Darby canine kidney cells (MDCK) permeability	-4.815
P-glycoprotein (Pgp)-inhibitor	0.321
P-glycoprotein (Pgp)-substrate	0.052
Human intestinal absorption (HIA)	0.006
20% bioavailability (F20%)	0.879
Distribution	
Plasma protein binding (PPB)	83.7%
Volume distribution (VD)	0.532
Blood–brain barrier (BBB) penetration	0.004
The fraction unbound in plasma (Fu)	16.4%
Metabolism	
CYP1A2-inhibitor	0.0
CYP1A2-substrate	0.011
CYP2C19-inhibitor	0.01
CYP2C19-substrate	0.017
CYP2C9-inhibitor	0.745
CYP2C9-substrate	0.035
CYP2D6-inhibitor	0.0
CYP2D6-substrate	0.617
CYP3A4-inhibitor	0.982
CYP3A4-substrate	0.252
Excretion	
Clearance	7.391
The half-life ($T_{1/2}$)	1.112
Toxicity	
hERG blockers	0.047
Human hepatotoxicity (H-HT)	0.65
Drug-induced liver injury (DILI)	0.76
Ames toxicity	0.562
Rat oral acute toxicity	0.271
Maximum recommended daily dose (FDAMDD)	0.476
Skin sensitization	0.962
Carcinogenicity	0.313
Eye corrosion	0.005
Eye irritation	0.81
Respiratory toxicity	0.246
Drug-likeness	
MCE-18	89.25
Lipinski rule	0.0

Table S14 cont.

Property	Value
Pfzer rule	0.0
Golden triangle	0.0
GSK rule	1.0

Table S15. ADMET and drug-likeness properties of BTL-A through the online prediction tool ADMETlab 3.0.

Property	Value
Absorption	
Caco-2 permeability	-4.988
Madin–Darby canine kidney cells (MDCK) permeability	-4.787
P-glycoprotein (Pgp)-inhibitor	0.139
P-glycoprotein (Pgp)-substrate	0.007
Human intestinal absorption (HIA)	0.015
20% bioavailability (F20%)	0.831
Distribution	
Plasma protein binding (PPB)	84.0%
Volume distribution (VD)	0.649
Blood–brain barrier (BBB) penetration	0.001
The fraction unbound in plasma (Fu)	16.9%
Metabolism	
CYP1A2-inhibitor	0.0
CYP1A2-substrate	0.212
CYP2C19-inhibitor	0.031
CYP2C19-substrate	0.002
CYP2C9-inhibitor	0.699
CYP2C9-substrate	0.038
CYP2D6-inhibitor	0.0
CYP2D6-substrate	0.648
CYP3A4-inhibitor	0.999
CYP3A4-substrate	0.991
Excretion	
Clearance	5.397
The half-life ($T_{1/2}$)	1.169
Toxicity	
hERG blockers	0.04
Human hepatotoxicity (H-HT)	0.381
Drug-induced liver injury (DILI)	0.595
Ames toxicity	0.339
Rat oral acute toxicity	0.232
Maximum recommended daily dose (FDAMDD)	0.472
Skin sensitization	0.876
Carcinogenicity	0.239
Eye corrosion	0.002
Eye irritation	0.766
Respiratory toxicity	0.258
Drug-likeness	
MCE-18	89.25
Lipinski rule	0.0

Table S15 cont.

Property	Value
Pfzer rule	0.0
Golden triangle	0.0
GSK rule	1.0

Table S16. ADMET and drug-likeness properties of BTL-B through the online prediction tool ADMETlab 3.0.

Property	Value
Absorption	
Caco-2 permeability	-4.733
Madin–Darby canine kidney cells (MDCK) permeability	-4.693
P-glycoprotein (Pgp)-inhibitor	0.17
P-glycoprotein (Pgp)-substrate	0.007
Human intestinal absorption (HIA)	0.004
20% bioavailability (F20%)	0.982
Distribution	
Plasma protein binding (PPB)	93.8%
Volume distribution (VD)	1.517
Blood–brain barrier (BBB) penetration	0.001
The fraction unbound in plasma (Fu)	5.4%
Metabolism	
CYP1A2-inhibitor	0.0
CYP1A2-substrate	0.003
CYP2C19-inhibitor	1.0
CYP2C19-substrate	0.998
CYP2C9-inhibitor	1.0
CYP2C9-substrate	0.017
CYP2D6-inhibitor	0.004
CYP2D6-substrate	0.754
CYP3A4-inhibitor	0.998
CYP3A4-substrate	0.97
Excretion	
Clearance	8.566
The half-life ($T_{1/2}$)	0.855
Toxicity	
hERG blockers	0.061
Human hepatotoxicity (H-HT)	0.42
Drug-induced liver injury (DILI)	0.504
Ames toxicity	0.402
Rat oral acute toxicity	0.293
Maximum recommended daily dose (FDAMDD)	0.582
Skin sensitization	0.945
Carcinogenicity	0.339
Eye corrosion	0.0
Eye irritation	0.49
Respiratory toxicity	0.485
Drug-likeness	
MCE-18	67.375
Lipinski rule	0.0

Table S16 cont.

Property	Value
Pfzer rule	0.0
Golden triangle	0.0
GSK rule	1.0

Table 17. ADMET and drug-likeness properties of territrem B through the online prediction tool ADMETlab 3.0.

Property	Value
Absorption	
Caco-2 permeability	-5.127
Madin–Darby canine kidney cells (MDCK) permeability	-4.756
P-glycoprotein (Pgp)-inhibitor	0.002
P-glycoprotein (Pgp)-substrate	0.002
Human intestinal absorption (HIA)	0.0
20% bioavailability (F20%)	0.722
Distribution	
Plasma protein binding (PPB)	91.6%
Volume distribution (VD)	1.673
Blood–brain barrier (BBB) penetration	0.003
The fraction unbound in plasma (Fu)	8.6%
Metabolism	
CYP1A2-inhibitor	0.0
CYP1A2-substrate	0.832
CYP2C19-inhibitor	0.969
CYP2C19-substrate	0.933
CYP2C9-inhibitor	0.931
CYP2C9-substrate	0.001
CYP2D6-inhibitor	0.0
CYP2D6-substrate	0.002
CYP3A4-inhibitor	0.553
CYP3A4-substrate	0.996
Excretion	
Clearance	6.379
The half-life ($T_{1/2}$)	1.506
Toxicity	
hERG blockers	0.052
Human hepatotoxicity (H-HT)	0.75
Drug-induced liver injury (DILI)	0.588
Ames toxicity	0.38
Rat oral acute toxicity	0.688
Maximum recommended daily dose (FDAMDD)	0.697
Skin sensitization	0.888
Carcinogenicity	0.942
Eye corrosion	0.016
Eye irritation	0.775
Respiratory toxicity	0.643
Drug-likeness	
MCE-18	131.182
Lipinski rule	0.0

Table S17 cont.

Property	Value
Pfzer rule	0.0
Golden triangle	1.0
GSK rule	1.0

References

[37] Afiyatullov, S.S.; Leshchenko, E.V.; Sobolevskaya, M.P.; et al. New 3- 2'(*R*)-hydroxybutyl -7-hydroxyphthalide from marine isolate of the fungus Penicillium claviforme. *Chem. Nat. Compd.* **2015**, 51, 111–115.

[39] Ellestad, G.A.; Evans, R.H., Jr.; Kunstmann, M.P. Some new terpenoid metabolites from an unidentified fusarium species. *Tetrahedron* **1969**, 25, 1323–1334.

[40] Zhang, P.; Bao, B.; Dang, H.T.; et al. Anti-inflammatory sesquiterpenoids from a sponge-derived fungus Acremonium sp. *J. Nat. Prod.* **2009**, 72, 270–275.

[41] Gui, R.Y.; Xu, L.; Kuang, Y.; et al. Chaetominine, (+)-alantrypinone, questin, isorhodoptilometrin, and 4-hydroxybenzaldehyde produced by the endophytic fungus *Aspergillus* sp YL-6 inhibit wheat (*Triticum aestivum*) and radish (*Raphanus sativus*) germination. *J. Plant Interact.* **2015**, 10, 87–92.

[42] Kittiwan, S.; Sasiphimol, S.; Phongphan, J.; Pakin, N.; Audomsak, C.; Nuttika, S.; Wiyada, M.; Thanaset, S.; Sarawut, T.; Pairot, M.; et al. Antiproliferative polyketides from fungus *Xylaria* cf. *Longipes* SWUF08-81 in different culture media. *Nat. Prod. Bioprosp.* **2024**, 14, 6.

[43] Li, Y.; Li, X.; Lee, U.; et al. A new radical scavenging anthracene glycoside, asperflavin ribofuranoside, and polyketides from a marine isolate of the fungus *Microsporum*. *Chem. Pharm. Bull.* **2006**, 54, 882–883.

[44] Chiang, C.C.; Huang, T.N.; Lin, Y.W.; et al. Enhancement of 4-acetylantroquinonol B production by supplementation of its precursor during submerged fermentation of *Antrodia cinnamomea*. *J. Agric. Food Chem.* **2013**, 61, 9160–9165.

[45] Haritakun, R.; Rachtawee, P.; Chanthaket, R.; et al. Butyrolactones from the fungus *Aspergillus terreus* BCC 4651. *Chem. Pharm. Bull.* **2010**, 58, 1545–1548.
